# In Situ Biomineralization Enhances Mitochondrial Transplantation to Differentiating Osteoclast Precursors for Suppressing Cancer-Induced Osteolysis

**DOI:** 10.34133/research.1410

**Published:** 2026-08-19

**Authors:** Yu Zhang, Changpeng Liu, Pengzhen Zhuang, Chengcheng Li, Huan Zhang, Yuanyuan Liu, Yu Chen, Wu Yang, Longxi Wu, Yawei Du, Wenguo Cui, Hongbo Zhang

**Affiliations:** ^1^ Department of Orthopaedics, Shanghai Key Laboratory for Prevention and Treatment of Bone and Joint Diseases, Shanghai Institute of Traumatology and Orthopaedics, Ruijin Hospital, Shanghai Jiao Tong University School of Medicine, 197 Ruijin 2nd Road, Shanghai 200025, PR China.; ^2^ Pharmaceutical Sciences Laboratory, Faculty of Science and Engineering, Åbo Akademi University, Turku 20520, Finland.; ^3^ Department of Orthopaedics, The First Affiliated Hospital of Soochow University, Soochow University, Suzhou 215000, China.; ^4^ State Key Laboratory of Water Environment Simulation, School of Environment, Beijing Normal University, Beijing 100875, China.; ^5^ Institute of Biomedicine, University of Turku, Turku 20014, Finland.; ^6^ Turku Bioscience Centre, University of Turku and Åbo Akademi University, Turku 20520, Finland.

## Abstract

Cancer-induced bone osteolysis is a common complication of multiple malignancies and may actively contribute to bone metastasis. Its core pathology is closely associated with mitochondrial metabolic dysfunction during osteoclast differentiation. In this study, a mitochondrial transplantation strategy based on in situ biomineralization (Mito@ZIF@RGD) was developed to overcome multiple delivery barriers in differentiating osteoclast precursor cells. A zeolitic imidazolate framework-8 (ZIF-8) shell was formed via the in situ self-assembly of Zn^2+^ and 2-methylimidazole on the mitochondrial membrane, thereby enhancing mitochondrial stability. Meanwhile, cyclic RGD (arginine–glycine–aspartic acid) peptides were coordinated with exposed Zn^2+^ sites on the outer shell to promote αvβ3-mediated uptake during osteoclast differentiation. Furthermore, the sustained Zn^2+^ release from the ZIF-8 biomineralization reshaped intracellular ionic homeostasis, thereby improving the durability of therapeutic efficacy following mitochondrial transplantation. In vitro experiments demonstrated that ZIF-8 encapsulation stabilized mitochondria and enabled sustained adenosine triphosphate production for more than 48 h. RGD modification improved cellular uptake efficiency by approximately 55% in differentiating osteoclast precursors, while the mildly acidic microenvironment triggered the coordinated release of mitochondria and Zn^2+^, effectively reducing intracellular reactive oxygen species levels and osteoclast formation. In vivo, Mito@ZIF@RGD treatment promoted the recovery of bone mineral density, suppressed osteoclast surface area formation by approximately 30%, and preserved bone microstructural integrity. Therefore, as a stable, specific, and durable mitochondrial transplantation platform modulating cellular metabolism during osteoclast differentiation, Mito@ZIF@RGD represents a stable and promising platform for the treatment of cancer-induced osteolysis and other metabolic imbalance-associated bone diseases.

## Introduction

Cancer-induced osteolysis is a common complication of malignancies such as breast and prostate cancers, affecting approximately 80% of patients with advanced disease progression and severely impairing prognosis and quality of life [1,2]. In addition, cancer-induced osteolysis can actively facilitate bone metastasis [3]; however, the underlying mechanisms and effective intervention strategies remain poorly defined. Among the key regulatory players, osteoclast precursor cells derived from the monocyte/macrophage lineage play a central role and represent critical therapeutic targets [4]. Emerging evidence indicates that tumor-associated factors, including lactate, receptor activator of nuclear factor-κB ligand (RANKL), and inflammatory cytokines, induce a distinct metabolic reprogramming during osteoclast differentiation characterized by concomitant up-regulation of oxidative phosphorylation and glycolysis, referred to as a “dual-high” metabolic phenotype [5,6]. This hypermetabolic state accelerates osteoclast differentiation and bone resorption. Although current therapeutic strategies, such as bisphosphonates and RANKL inhibitors [7], can partially suppress bone resorption, they primarily target downstream effector processes and fail to directly correct mitochondrial dysfunction and metabolic imbalance during osteoclast differentiation [8]. Therefore, the development of more effective metabolic intervention strategies targeting differentiating osteoclast precursor cells is of critical importance for suppressing cancer-induced osteolysis at its source.

Mitochondrial transplantation has emerged as a promising cellular metabolic intervention strategy, capable of restoring energy metabolism and alleviating oxidative stress through the direct delivery of healthy mitochondria [9,10]. In cancer-induced osteolysis, however, the pathological “dual-high” metabolic state during osteoclast differentiation is characterized by concurrently elevated oxidative phosphorylation and glycolysis. This state imposes excessive burden on the mitochondrial electron transport chain, leading to hyperpolarization-associated electron leakage and sustained overproduction of reactive oxygen species (ROS), a process that is difficult to rapidly reverse [5,6]. Under these conditions, mitochondrial transplantation represents one of the most effective strategies currently available for targeting metabolic dysregulation at its origin during osteoclast differentiation. Nevertheless, achieving efficient mitochondrial transplantation in osteoclast precursors under cancer-induced osteolysis remains challenged by multiple barriers, including insufficient stability, limited cell specificity, and poor durability [11,12]. Healthy mitochondria are intrinsically unstable within pathological microenvironments and may be prematurely compromised by the dual-high metabolic phenotype. Moreover, the lack of intrinsic affinity for differentiating osteoclast precursor cells in vivo severely limits mitochondrial delivery efficiency. Therefore, modification strategies that enhance mitochondrial stability and targeting specificity are essential for enabling efficient mitochondrial transplantation in differentiating osteoclast precursor cells under cancer-induced osteolysis. More critically, the long-term sustainability of metabolic intervention following conventional mitochondrial transplantation remains uncertain. Recent studies have demonstrated that mitochondrial functional homeostasis depends not only on mitochondrial abundance and structural integrity but also on the stability of the intracellular ionic microenvironment [13]. Among these factors, zinc and calcium ion homeostasis play central regulatory roles in maintaining mitochondrial membrane potential, supporting energy metabolism, and suppressing ROS-associated oxidative stress [13,14]. Given the pivotal involvement of metal ion dysregulation in tumor pathology, particularly in breast cancer and other malignancies, zinc depletion disrupts mitochondrial membrane potential maintenance and oxidative phosphorylation, resulting in persistent ROS accumulation and subsequent activation of osteoclast-related signaling pathways such as NFATc1, thereby promoting excessive osteoclast differentiation [15,16]. Consequently, under cancer-induced osteolysis, transplantation of healthy mitochondria alone is insufficient to achieve sustained metabolic correction, highlighting the urgent need for a synergistic therapeutic strategy that concurrently preserves the ionic homeostasis required for continuous mitochondrial functional operation following transplantation.

In recent years, metal ion-based biomineralization materials have provided new strategies to address the long-standing challenge of the dissociation between delivery efficiency and functional maintenance in mitochondrial transplantation. Among these materials, metal–organic frameworks have attracted increasing attention as versatile carriers for bioactive cargo delivery and cellular ionic homeostasis regulation, owing to their excellent biocompatibility, tunable composition, and controllable release behavior [17–19]. Previous studies have demonstrated that metal–organic framework materials can be broadly applied in the regulation of tissue metabolism and inflammation [20,21]. In particular, zeolitic imidazolate framework-8 (ZIF-8) has been shown to enable biomineralization of proteins, enzymes, and genetic materials under mild conditions [22–24], while achieving the coordinated release of biomacromolecules and Zn^2+^ in acidic environments, highlighting its potential advantages in cargo protection and ion regulation. However, conventional ZIF-8-based delivery strategies primarily address cargo preservation and fail to simultaneously satisfy 2 critical requirements for mitochondrial transplantation: cell-specific delivery and sustained posttransplantation functional maintenance [24]. Recently, Zhang’s group [9,10] reported a ZIF-8-encapsulated mitochondrial transplantation strategy that achieved long-term preservation of mitochondrial structural and functional integrity in vitro and enabled pH-responsive release under acidic conditions. Nevertheless, this approach mainly relies on passive cellular uptake and lacks active lesion targeting and efficient internalization, thereby limiting its applicability in complex pathological microenvironments. Notably, the hierarchical coordination structure formed during mitochondrial biomineralization with ZIF-8 provides accessible sites for surface ligand modification [25]. In particular, given the expression and accumulation of integrin αvβ3 on differentiating osteoclast precursor cells [26–28], the introduction of corresponding targeting peptides offers the potential to achieve precise localization and efficient uptake of ZIF-8-encapsulated mitochondria by differentiating osteoclast precursor cells. Importantly, however, achieving long-term therapeutic efficacy of mitochondrial transplantation in cancer-induced osteolysis cannot rely solely on improved delivery efficiency. A key remaining challenge lies in maintaining the ionic homeostasis required for sustained mitochondrial function following transplantation. In this context, the Zn^2+^ released from ZIF-8 after mitochondrial transplantation plays a critical role in restoring zinc ion imbalance in tumor-conditioned osteoclast precursors [13,16], thereby supporting the continuous functional operation of transplanted mitochondria. Therefore, the development of an efficient mitochondrial transplantation strategy based on in situ biomineralization at the mitochondrial surface, combined with cell-specific targeting, holds promise for restoring energy metabolism, alleviating ROS accumulation, and suppressing osteoclast activation in cancer-induced osteolysis. Such an approach offers a new avenue for metabolic reprogramming-driven restoration of bone dynamic homeostasis.

**Fig. 1.**
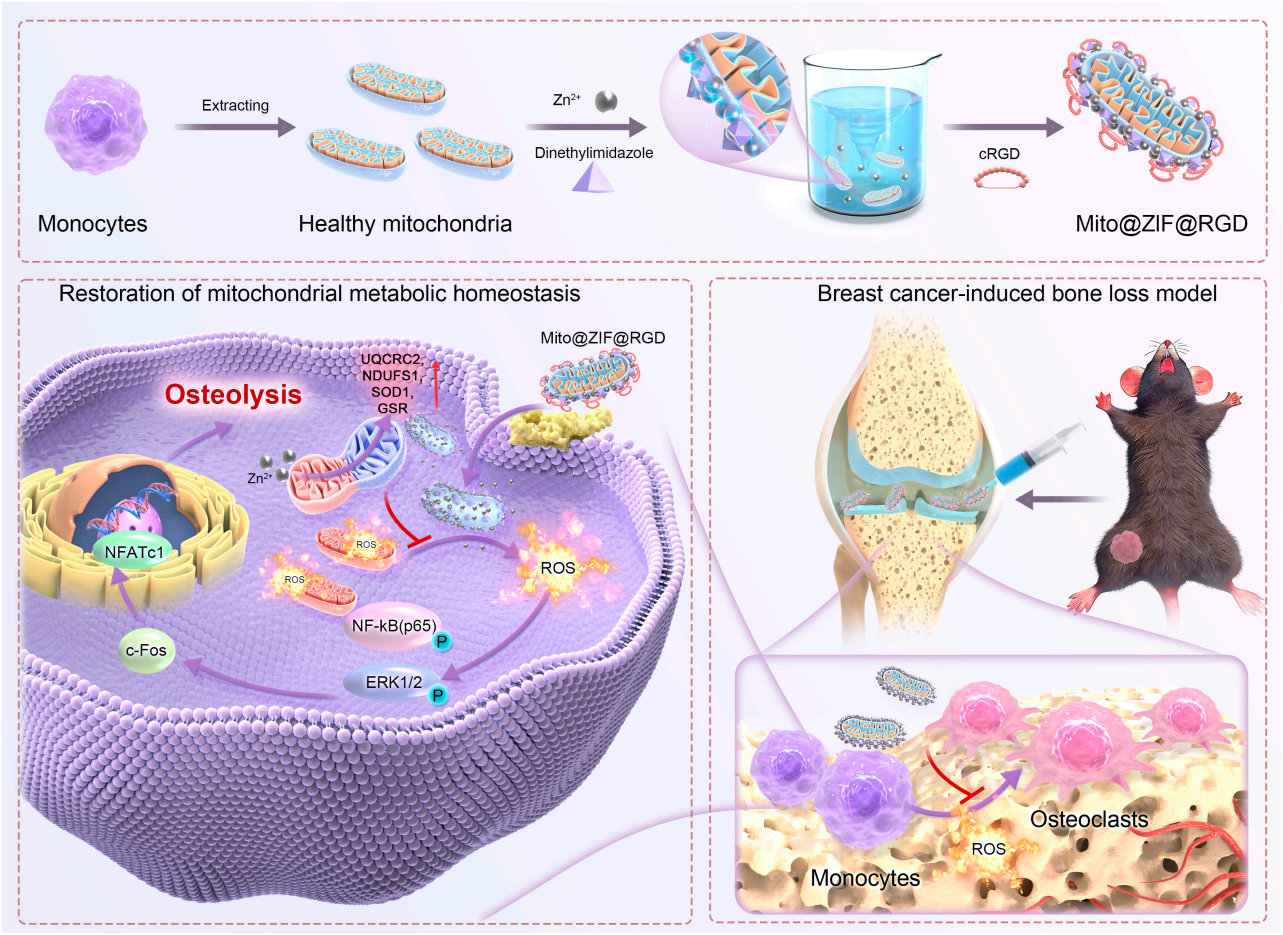
Extraction and biomineralization of healthy mitochondria for restoring mitochondrial metabolic homeostasis in breast cancer-induced osteolysis.

In this work, we developed an in situ biomineralization-enabled mitochondrial transplantation strategy to promote efficient mitochondrial delivery into differentiating osteoclast precursor cells, generating engineered homologous mitochondria termed Mito@ZIF@RGD for targeted intervention in cancer-induced osteolysis (Fig. 1). In this system, healthy mitochondria were employed as the cargo, and Zn^2+^ and 2-methylimidazole were in situ self-assembled on the mitochondrial surface to form a ZIF-8 mineralized shell (Mito@ZIF). Subsequently, cyclic RGD (arginine–glycine–aspartic acid) (cRGD) peptides were coordinated with accessible outer coordination sites (exposed Zn^2+^) to achieve αvβ3-integrin-directed bone targeting and uptake of differentiating osteoclast precursor cells, resulting in the Mito@ZIF@RGD nanoplatform. This platform significantly prolonged mitochondrial functional preservation in vitro and enabled specific enrichment and efficient uptake of both Zn^2+^ and functional mitochondria by differentiating osteoclast precursor cells, thereby reversing mitochondrial metabolic imbalance and suppressing breast cancer-induced ROS elevation and subsequent osteoclast activation. In an orthotopic breast cancer-induced bone osteolysis model, Mito@ZIF@RGD effectively attenuated bone destruction and preserved bone structural integrity. Collectively, this study establishes, for the first time, an in situ biomineralization-based strategy that enhances mitochondrial transplantation to correct metabolic dysfunction during osteoclast differentiation, enabling differentiating osteoclast precursor-specific metabolic reprogramming and providing a new mitochondrial transplantation paradigm for sustained metabolic intervention in cancer-induced osteolysis.

## Results

### Preparation and characterization of mitochondrial delivery nanoplatforms

**Fig. 2.**
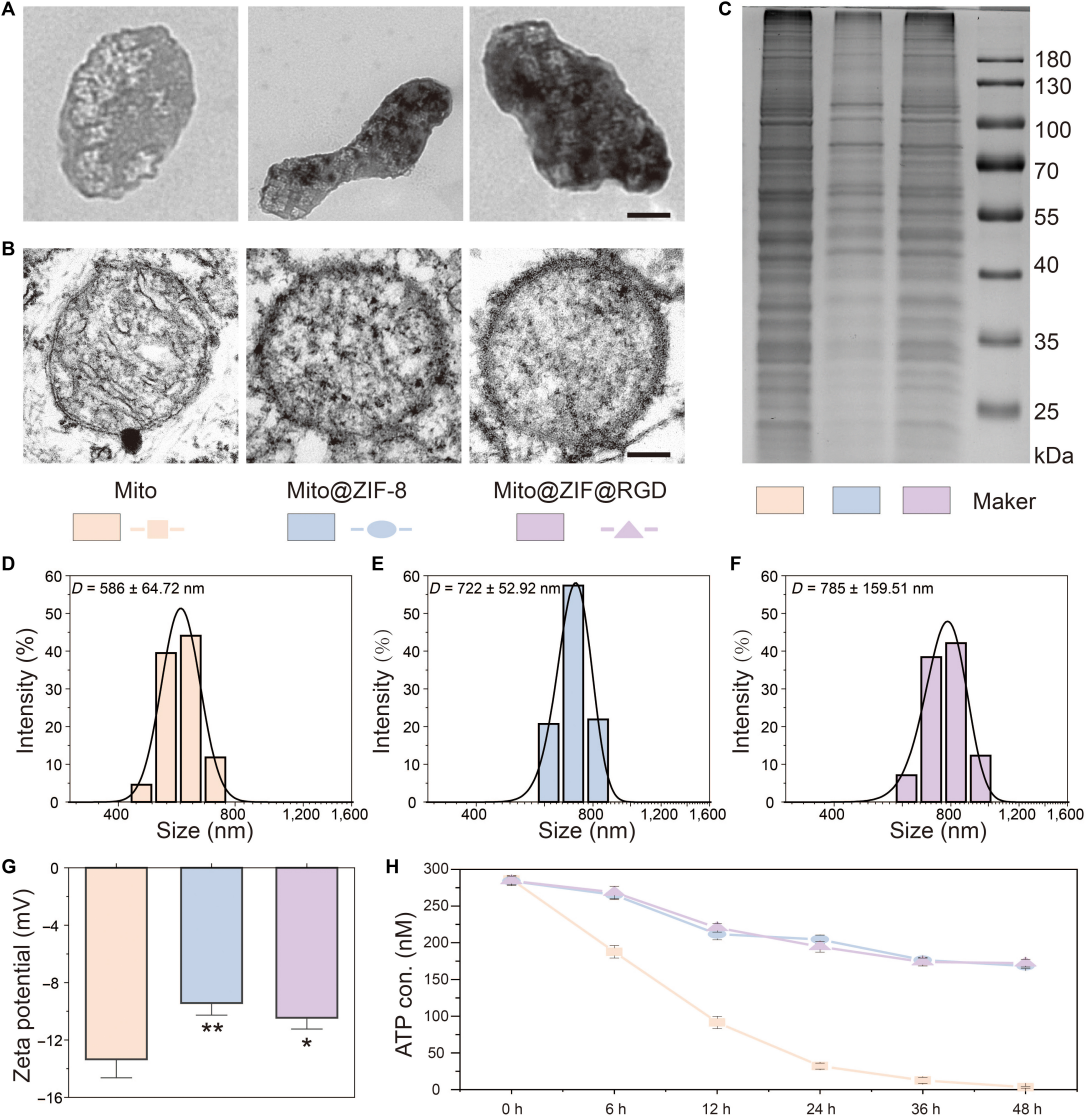
Characterization of isolated mitochondria and preservation of adenosine triphosphate (ATP) activity. (A) Transmission electron microscopy (TEM) image of isolated mitochondria. (B) Representative ultrathin-sectioned TEM images of isolated mitochondria, Mito@ZIF-8, and Mito@ZIF@RGD after encapsulation. (C) Coomassie Brilliant Blue staining of mitochondrial proteins. (D to F) Particle size distribution of different nanoparticle formulations. (G) Zeta potential of the corresponding nanoparticles. (H) Time-dependent ATP production assay of mitochondria. Scale bars represent 200 μm, *n* = 3, *P < 0.05, **P < 0.01.

The mitochondrial delivery system (Mito@ZIF@RGD) was successfully constructed via in situ self-assembly of ZIF-8 on the surface of healthy mitochondria, followed by RGD peptide modification. Transmission electron microscopy (TEM) images (Fig. 2A) showed that the extracted mitochondria exhibited a typical double-membrane structure with clear cristae, and the ZIF-8 shell formed a uniform protective layer around the mitochondria (Fig. 2B). Coomassie Brilliant Blue staining confirmed the integrity of mitochondrial protein components in the prepared nanocomplexes (Fig. 2C).

Dynamic light scattering analysis revealed the particle size distribution of the nanoplatforms (Fig. 2D to F). The bare mitochondria had an average diameter of 586 ± 64.72 nm, which increased to 722 ± 52.92 nm after ZIF-8 encapsulation (Mito@ZIF-8) and further to 785 ± 159.51 nm following RGD modification (Mito@ZIF@RGD), indicating successful coating and functionalization. Zeta potential measurements (Fig. 2G) showed a negative surface charge of the nanocomplexes, which is favorable for nanocomplex stability in biological environments. Zeta potential measurements also revealed that ZIF-8 mineralization markedly improved the long-term stability and preservation of mitochondria over 7 d (Fig. S1). Adenosine triphosphate (ATP) production assays (Fig. 2H) demonstrated that Mito@ZIF@RGD maintained mitochondrial bioactivity over 48 h, with sustained ATP generation, whereas bare mitochondria showed a rapid decline in activity, confirming the protective effect of the ZIF-8 shell. Meanwhile, the Zn^2+^ release behavior of Mito@ZIF@RGD was evaluated in buffer solutions at pH 6.5 and pH 7.4, as shown in Fig. S2. Under mildly acidic conditions mimicking the tumor-conditioned microenvironment of osteoclast precursor cells, a rapid release of Zn^2+^ was observed within the first 6 h, with cumulative release reaching approximately 75% within 24 h. In contrast, Mito@ZIF@RGD largely maintained its nanoparticle structural integrity in neutral buffer, exhibiting minimal Zn^2+^ release over 24 h.

### Cytocompatibility of Mito@ZIF@RGD nanoplatforms

**Fig. 3.**
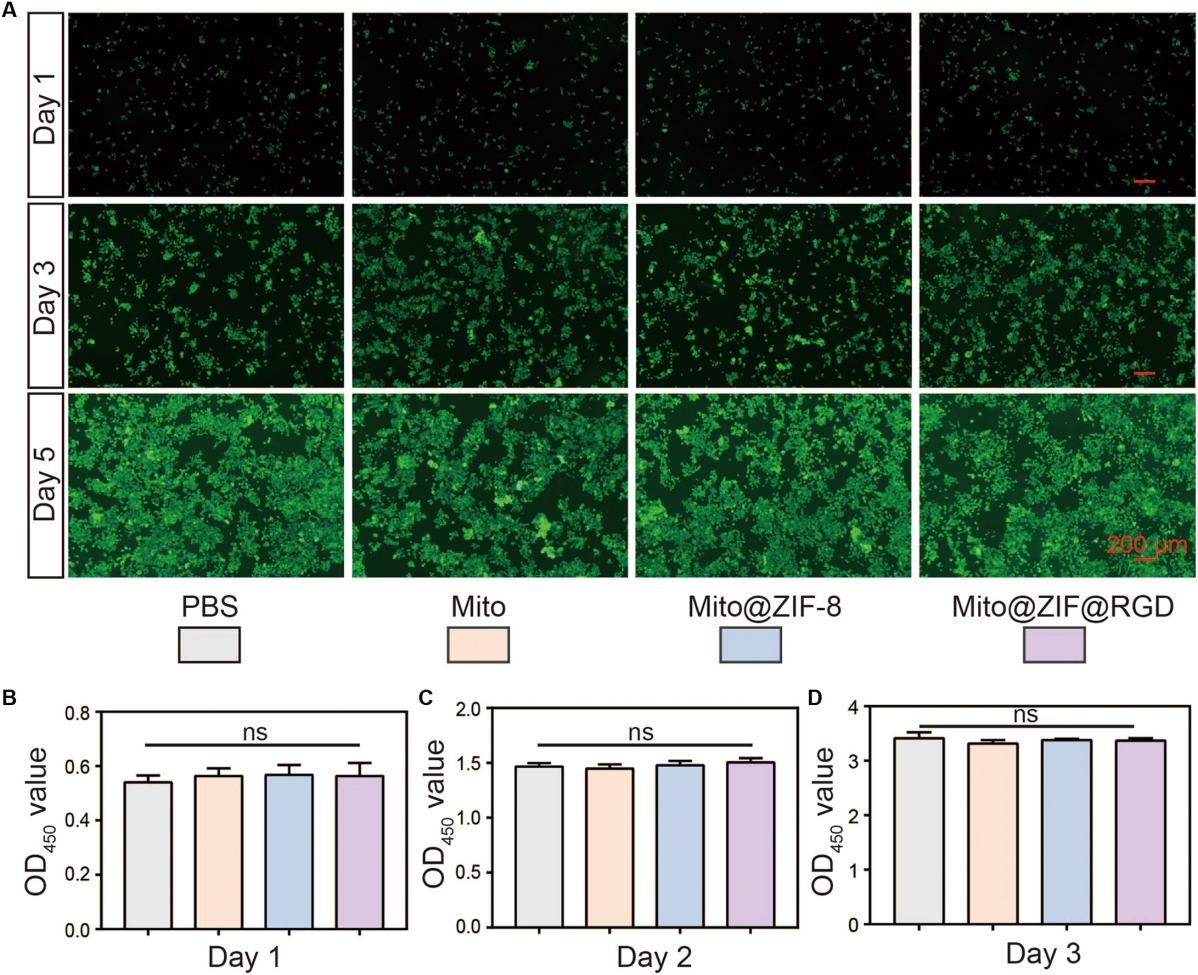
Biocompatibility evaluation of the Mito@ZIF@RGD nanoplatform. (A) Live/dead staining of cells after 1, 3, and 5 d of treatment. (B to D) Cell viability assessed by Cell Counting Kit-8 assay after 1, 2, and 3 d of treatment. Scale bars represent 200 μm, *n* = 3. OD_450,_ optical density at 450 nm; ns, not significant.

To verify the biosafety of the mitochondrial nanoplatforms, comprehensive cytocompatibility evaluations were performed using live/dead cell staining and the Cell Counting Kit-8 (CCK-8) assay on RAW264.7 cells (osteoclast precursor cells), which are the key target cells for breast cancer-induced bone osteolysis intervention. For live/dead staining (Fig. 3A), cells were cocultured with phosphate-buffered saline (PBS) (negative control), bare mitochondria (Mito), Mito@ZIF-8, and Mito@ZIF@RGD for 1, 3, and 5 d, respectively. Microscopic observations showed that across all time points, the majority of cells in each group exhibited bright green fluorescence, indicating intact cell membranes and normal viability. Red fluorescence signals (representing dead cells) were barely detectable in all experimental groups, with the dead cell ratio consistently below 5%—a level equivalent to the PBS control group. Even after 5 d of long-term coculture, Mito@ZIF@RGD did not induce obvious cell death, confirming the system’s excellent long-term biocompatibility. The CCK-8 assay further quantified cell proliferation activity (Fig. 3B to D). The optical density values at 450 nm (an indicator of viable cell number) showed a gradual upward trend in all groups from day 1 to day 3, consistent with the normal proliferation rhythm of RAW264.7 cells. At days 1, 2, and 3, no statistically significant differences in absorbance were observed among the PBS, Mito, Mito@ZIF-8, and Mito@ZIF@RGD groups. These results indicate that the nanoparticles, including the ZIF-8 coating and RGD modification, had no detectable adverse effects on cell viability or proliferation.

### Targeted phagocytosis of Mito@ZIF@RGD by differentiating osteoclast precursor cells

To assess the targeted delivery efficiency of Mito@ZIF@RGD to differentiating osteoclast precursor cells, we first evaluated the time-dependent expression of integrin αVβ3 in RAW264.7 cells following cotreatment with E0771 cells and RANKL. Compared with the PBS group, both gene and protein expression levels of integrin αVβ3 gradually increased during osteoclast differentiation of RAW264.7 cells at days 1, 2, 3, and 5 (Fig. S3). Notably, a significant up-regulation of αVβ3 expression was observed at days 2 and 3 after stimulation (Fig. S3A to C). To further evaluate whether RGD modification enhances the cellular uptake of nanoparticles in differentiating RAW264.7 cells, free RGD peptide and an integrin αvβ3 blocking agent [Cyclo(RGDfC)] were used to pretreat cells prior to nanoparticle administration. In addition, nontargeted peptide-modified nanoparticles (Mito@ZIF@RGE) and primary bone marrow mesenchymal stem cells with low αvβ3 expression were included as controls. Compared with these groups, Mito@ZIF@RGD nanoparticles exhibited markedly enhanced uptake by RAW264.7 cells (Fig. S4). Furthermore, to investigate the lysosomal fate of internalized mitochondria, differentiating RAW264.7 cells were coincubated with Mito@ZIF@RGD for 0.5 and 4 h, followed by confocal microscopy analysis. At 0.5 h, mitochondria labeled with MitoTracker Green showed pronounced colocalization with LysoTracker-labeled lysosomes. In contrast, at 4 h, partial mitochondrial escape from lysosomes into the cytoplasm was observed (Fig. S5).

**Fig. 4.**
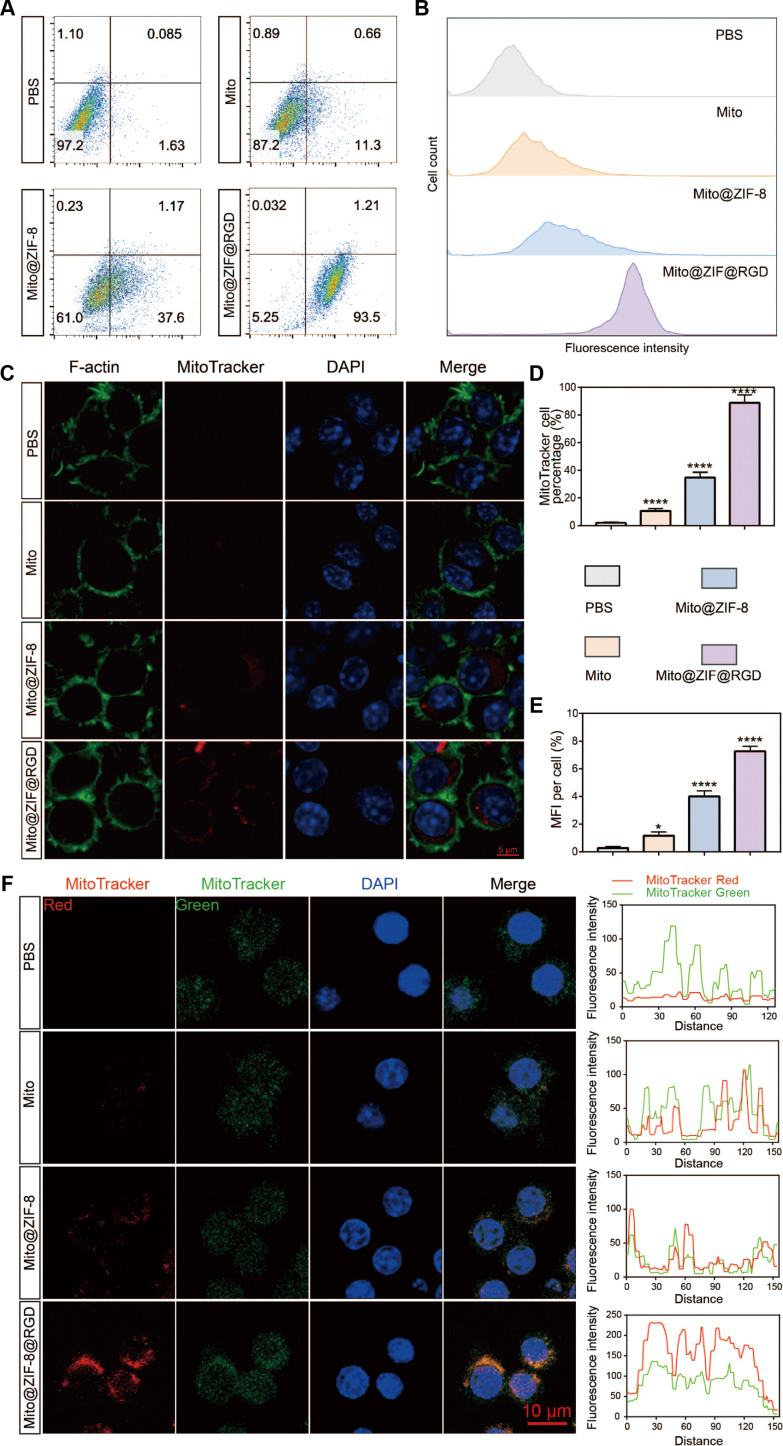
Targeted uptake of nanoparticles by RAW264.7 cells treated with RANKL. (A and B) Flow cytometry analysis of nanoparticle uptake by RAW264.7 cells. (C) Confocal laser scanning microscopy images showing nanoparticle internalization by RAW264.7 cells (F-actin [green], Mitochondria [red], and Nucleus [blue]). Scale bars represent 5 μm. (D) Quantification of nanoparticle uptake based on flow cytometry data. (E) Quantitative analysis of confocal fluorescence intensity. (F) Intracellular distribution and colocalization analysis of exogenous mitochondria (MitoTracker Red) and endogenous mitochondrial (MitoTracker Green) networks, 4′,6-diamidino-2-phenylindole (DAPI): nuclei, Scale bars represent 10 μm. *n* = 3, *P < 0.05, ****P < 0.0001.

Based on this findings, flow cytometry (FCM) and confocal laser scanning microscopy (CLSM) were employed to evaluate cellular uptake, with representative results shown in Fig. 4. FCM dot plots (Fig. 4A) and fluorescence intensity histograms (Fig. 4B) revealed minimal background fluorescence in the PBS group, confirming no nonspecific interference. The bare Mito group exhibited weak fluorescence signals, corresponding to a phagocytosis rate of 11.83% ± 3.1%, while the nontargeted Mito@ZIF-8 group showed an increased phagocytosis rate of 37.71% ± 2.8%. In contrast, the Mito@ZIF@RGD group displayed a marked shift toward higher fluorescence intensity, with a phagocytosis rate of 94.35% ± 1.5% that was significantly higher than both the Mito and Mito@ZIF-8 groups (Fig. 4D).

CLSM images (Fig. 4C) further validated these findings, with triple staining (MitoTracker Red for mitochondria, Alexa Fluor 488–phalloidin for F-actin, and 4′,6-diamidino-2-phenylindole for nuclei) showing sparse, weak red fluorescence in the Mito and Mito@ZIF-8 groups, while the Mito@ZIF@RGD group exhibited bright, uniform red fluorescence colocalized with cytoplasmic F-actin. Quantitative analysis of the CLSM fluorescence intensity (Fig. 4E) showed that the mean fluorescence intensity per cell in the Mito@ZIF@RGD group was approximately 7.2%, which was more than 6-fold higher than that in the Mito group and approximately 1.8-fold higher than that in the Mito@ZIF-8 group. These results support the enhanced cellular targeting of Mito@ZIF@RGD toward RAW264.7 cells following RGD modification.

To evaluate the effect of RGD modification on the uptake of mitochondrial nanoparticles by differentiating RAW264.7 cells, CLSM was used to visualize intracellular distribution of exogenous mitochondria (red) and endogenous mitochondria (green) (Fig. 4F). Compared with the PBS group, the Mito group exhibited weak red fluorescence signals within cells, whereas the Mito@ZIF-8 group showed markedly enhanced red fluorescence, indicating improved cellular uptake mediated by ZIF-8 encapsulation. Notably, the Mito@ZIF@RGD group displayed the strongest intracellular red fluorescence, suggesting that RGD modification further markedly enhances nanoparticle internalization in differentiating RAW264.7 cells. Line scan analysis further confirmed these findings (Fig. 4F, right panel). In the Mito@ZIF@RGD group, higher codistribution of MitoTracker Red and MitoTracker Green signals was observed, whereas more dispersed signal patterns were detected in the other groups. These results suggest that RGD modification promotes intracellular delivery of exogenous mitochondria and may facilitate their interaction or fusion with endogenous mitochondrial networks.

### Remodeling zinc ion homeostasis in RAW264.7 cells

**Fig. 5.**
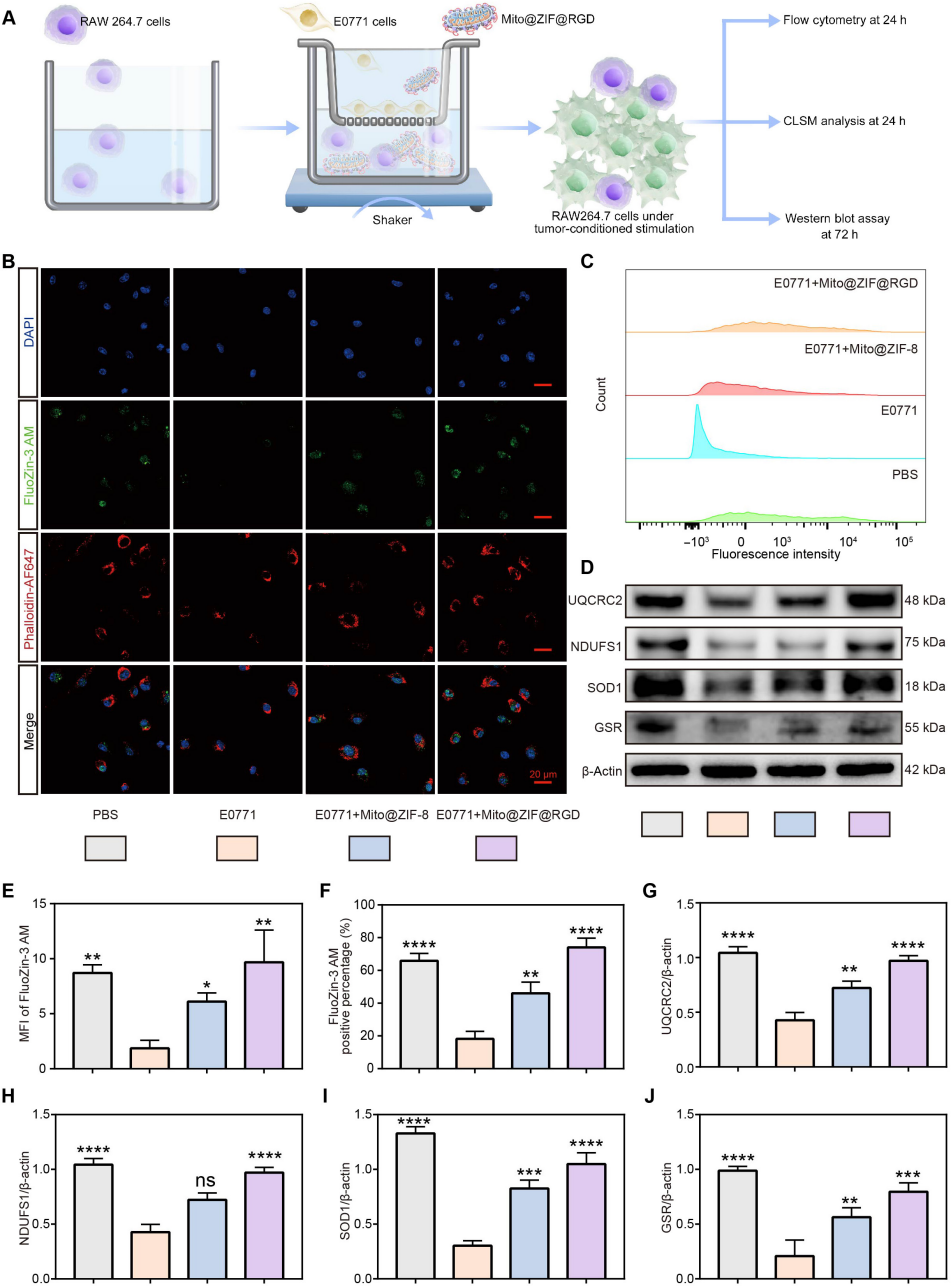
Assessment of zinc ion homeostasis restoration in RAW264.7 cells after breast cancer conditioning. (A) Coculture system of E0771 cells and RAW264.7 cells under Mito@ZIF@RGD treatment. (B) Confocal laser scanning microscopy images of intracellular Zn^2+^ in FluoZin-3 AM-labeled RAW264.7 cells (F-actin [red], Zn^2+^ [FluoZin-3 AM, green], and Nucleus [blue]). (C) Flow cytometry analysis of intracellular Zn^2+^ levels in FluoZin-3 AM-labeled RAW264.7 cells. (D) Representative Western blot images of proteins UQCRC2, NDUFS1, superoxide dismutase 1 (SOD1), glutathione reductase (GSR), and β-actin. (E) Quantification of intracellular Zn^2+^ fluorescence intensity based on confocal microscopy images. (F) Quantitative analysis of intracellular Zn^2+^ levels derived from flow cytometry data. (G to J) Quantitative analysis of UQCRC2, NDUFS1, SOD1, GSR, and β-actin protein expression. Scale bars represent 20 μm, *n* = 3, *P < 0.05, **P < 0.01, ***P < 0.001, ****P < 0.0001. ns, not significant.

Accumulating evidence indicates that zinc ion efflux and intracellular zinc loss under inflammatory conditions constitute a critical driving force for osteoclast differentiation. As zinc ion homeostasis is fundamental for the activity of numerous intracellular enzymes, chronic inflammation established by tumor cells within the bone microenvironment accelerates zinc depletion in osteoclast precursor cells, thereby facilitating osteoclast differentiation and the formation of metastatic osteolytic lesions. Based on this premise, a coculture model based on E0771 cells and RAW264.7 cells was established (Fig. 5A), intracellular zinc levels in RAW264.7 cells conditioned by the murine breast cancer cell line E0771 were evaluated using CLSM and FCM, and the restorative effect of the nanoplatform on zinc homeostasis was further assessed. As shown in Fig. 5B and E, compared with normally cultured cells (PBS group), E0771 cell coculture group (E0771 group) markedly promoted intracellular zinc loss in RAW264.7 cells. Quantitative fluorescence analysis revealed a 78.64% reduction in zinc-associated fluorescence intensity in the E0771 group. Notably, treatment with the nanoplatform partially restored intracellular zinc levels, with the Mito@ZIF-8@RGD nanoplatform (E0771+Mito@ZIF-8@RGD group) inducing a zinc recovery level slightly higher than that observed in the PBS group. To further validate the zinc restoration effect, flow cytometric analysis was performed. Consistent with the confocal microscopy results, RAW264.7 cells treated with E0771+Mito@ZIF-8 and E0771+Mito@ZIF@RGD, as labeled by FluoZin-3 AM, exhibited significantly increased intracellular zinc fluorescence intensities compared with the E0771 group, with increases of 27.77% and 55.80%, respectively (Fig. 5C and F). Among these, the fluorescence intensity of the E0771+Mito@ZIF@RGD group was slightly higher than that of the PBS group; however, no statistically significant difference was observed.

We subsequently investigated whether alterations in intracellular zinc homeostasis among different treatment groups influenced the expression of key enzymes responsible for sustaining mitochondrial function following transplantation in RAW264.7 cells. As shown in Fig. 5D, compared with RAW264.7 cells cocultured with E0771 cells, treatment with Mito@ZIF-8@RGD nanoparticles markedly increased the protein expression levels of UQCRC2, NDUFS1, superoxide dismutase 1 (SOD1), and glutathione reductase (GSR), restoring them to levels nearly comparable to those of the healthy control group. In contrast, the nontargeted Mito@ZIF-8 treatment resulted in only a modest recovery of these proteins. Quantitative protein analysis further confirmed a consistent trend across all groups (Fig. 5G to J).

### Regulation of metabolism and differentiation of differentiating osteoclast precursor cells by Mito@ZIF@RGD

**Fig. 6.**
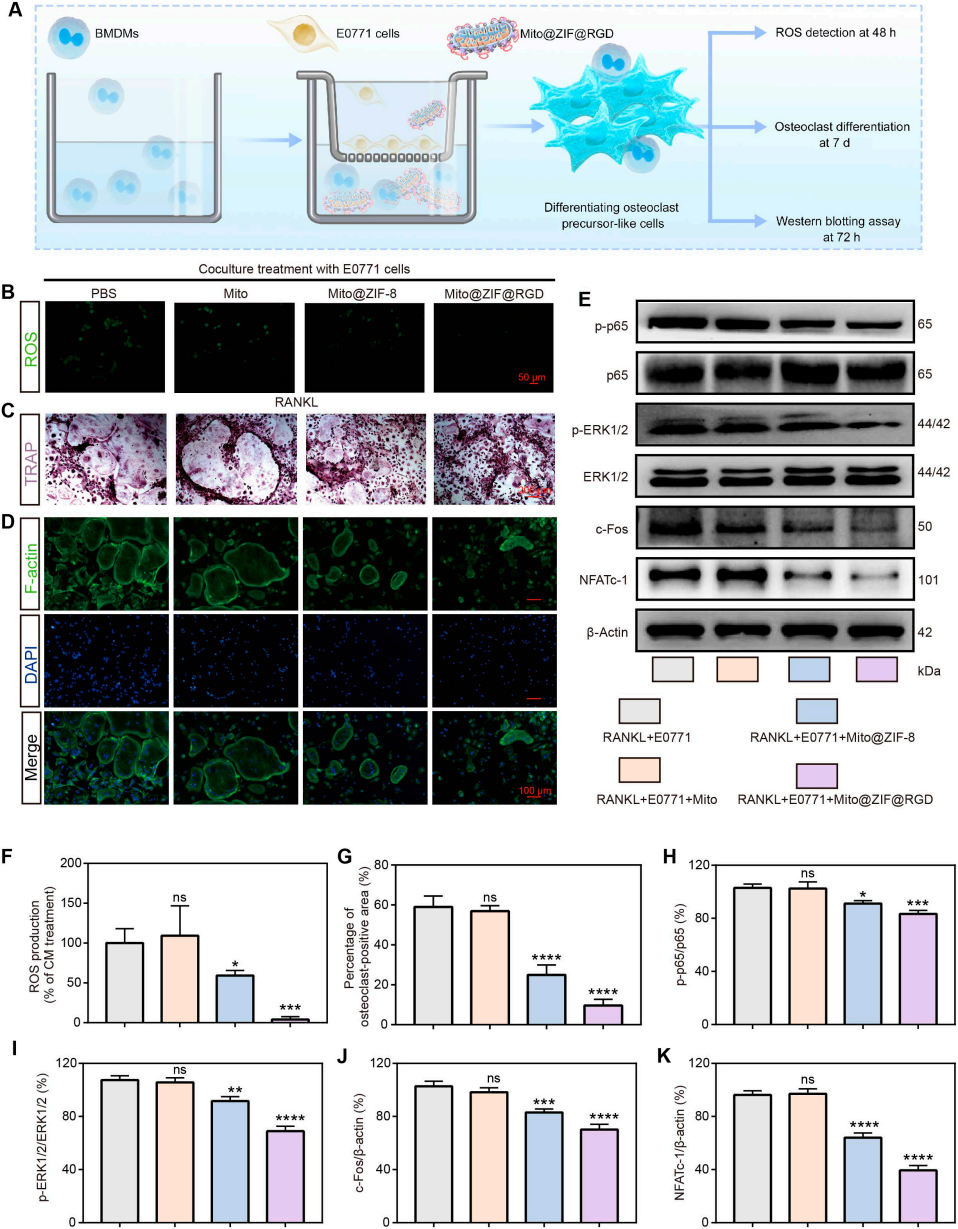
Osteoclast differentiation of cancer-conditioned osteoclast precursor cells following each group treatment. (A) Coculture system of E0771 cells and bone marrow-derived macrophages (BMDMs) under Mito@ZIF@RGD treatment. (B) Intracellular reactive oxygen species (ROS) staining. (C) Tartrate-resistant acid phosphatase (TRAP) staining for osteoclast differentiation. (D) Cytoskeletal staining of differentiated osteoclasts showing F-actin (green) and nuclei (blue). (E) Western blot analysis of p-p65, p65, p-ERK1/2, ERK1/2, c-Fos, NFATc1, and β-actin expression. (F) Quantification of relative ROS levels. (G) Percentage of TRAP-positive area induced during osteoclast differentiation. (H to K) Quantitative analysis of protein expression levels shown in (D). Each group: Osteoclast precursors stimulated with receptor activator of nuclear factor-κB ligand (RANKL) under E0771 coculture conditions (RANKL + E0771); cells treated with bare mitochondria (RANKL + E0771 + Mito); cells treated with zeolitic imidazolate framework-8 (ZIF-8)-encapsulated mitochondria (RANKL + E0771 + Mito@ZIF-8); and cells treated with RGD (arginine–glycine–aspartic acid)-modified Mito@ZIF (RANKL + E0771 + Mito@ZIF@RGD). Scale bars represent 20, 100, and 200 μm, respectively, *n* = 3, *P < 0.05, **P < 0.01, ***P < 0.001, ****P < 0.0001. ns, not significant.

To investigate the effects of homologous healthy mitochondria and zinc ions on metabolic reprogramming of tumor-induced osteoclast precursor cells, we established an in vitro coculture system using E0771 breast cancer cells and bone marrow-derived macrophages (BMDMs) to evaluate the impact of Mito@ZIF@RGD on cellular metabolism and osteoclast differentiation (Fig. 6A). The JC-1 staining results after 24 h of nanoparticle treatment (Fig. S6) showed that, compared with the control group, RANKL treatment increased JC-1 aggregate (red) fluorescence and reduced JC-1 monomer (green) signal, indicating an increase in mitochondrial membrane potential associated with early osteoclast differentiation-related metabolic activation. In contrast, cotreatment with RANKL and E0771 cells resulted in a decrease in JC-1 aggregate (red) signal and a relative increase in JC-1 monomer (green) signal, suggesting mitochondrial membrane potential perturbation potentially associated with tumor cell-derived microenvironmental stress. Under different intervention conditions, all treatment groups exhibited varying degrees of regulatory effects on JC-1 fluorescence distribution. Specifically, Matched Zn^2+^, ZIF@RGD, Mito@ZIF-8, Mito-RGD, and inactive Mito@ZIF@RGD groups all showed a reduction in JC-1 monomer (green) signal and a partial recovery of JC-1 aggregate (red) signal compared with the RANKL + E0771 + Mito group. Among all treatment groups, Mito@ZIF@RGD exhibited the most pronounced tendency toward restoration of JC-1 aggregate signal and reduction of monomer signal, suggesting a potential role of zinc-assisted mitochondrial delivery in maintaining mitochondrial membrane potential homeostasis.

Intracellular ROS analysis after 3 d of nanoparticle treatment (Fig. 6B and F and Fig. S7) showed that, compared with the RANKL group, RANKL + E0771 group and the RANKL + E0771 + Mito group, ROS levels were reduced in the matched zinc ion group (RANKL + E0771 + matched Zn^2+^, 25 μM), RANKL + E0771 + ZIF@RGD group, RANKL + E0771 + Mito@ZIF-8 group, RANKL + E0771 + inactive Mito@ZIF@RGD group, and RANKL + E0771 + Mito-RGD group. These results suggest that both zinc ions and functional exogenous mitochondria contribute to alleviating oxidative stress in osteoclast precursor cells. Notably, the most pronounced ROS reduction was observed in the Mito@ZIF@RGD-treated group, indicating that RGD–αvβ3-mediated delivery of healthy mitochondria effectively attenuates excessive oxidative stress in differentiating osteoclast precursors. Consistently, osteoclast differentiation assays (Fig. 6 and Fig. S8) further confirmed the synergistic effects of zinc ions and functional mitochondria. Compared with the RANKL group, RANKL + E0771 group, and the RANKL + E0771 + Mito group, all treatment groups—including RANKL + E0771 + matched Zn^2+^, ZIF@RGD, Mito@ZIF-8, inactive Mito@ZIF@RGD, and Mito-RGD—partially suppressed osteoclast formation. However, Mito@ZIF@RGD exhibited the strongest inhibitory effect on tartrate-resistant acid phosphatase (TRAP)-positive multinucleated osteoclast formation (Fig. 6C and Fig. S8A). This was further supported by cytoskeletal staining and osteoclast area quantification (Fig. 6D and G and Fig. S8B), Western blot analysis (Fig. 6E and H to K), and reverse transcription quantitative polymerase chain reaction results (*Acp5*, *Ctsk*, and *Nfatc1* expression; Fig. S8C to E), collectively indicating a potential synergistic role of zinc ions and functional mitochondrial delivery in suppressing osteoclastogenic signaling. Consistent with these findings, the bone resorption pit assay was performed to assess osteoclast resorptive activity. Compared with the RANKL+E0771 group, Mito@ZIF@RGD treatment reduced the resorption areas, as shown by representative images and quantitative analysis (Fig. S9A and B). These results suggest that Mito@ZIF@RGD may alleviate tumor-enhanced osteoclast-mediated bone resorption. Overall, these results suggest that Mito@ZIF@RGD is associated with reduced ROS levels and attenuated the enhanced osteoclast resorptive activity under tumor-stimulated conditions. The coordinated effects observed in ROS production, osteoclast formation, and related molecular markers indicate a potential regulatory role of zinc ions and functional mitochondrial delivery in modulating osteoclastogenic activity.

**Fig. 7.**
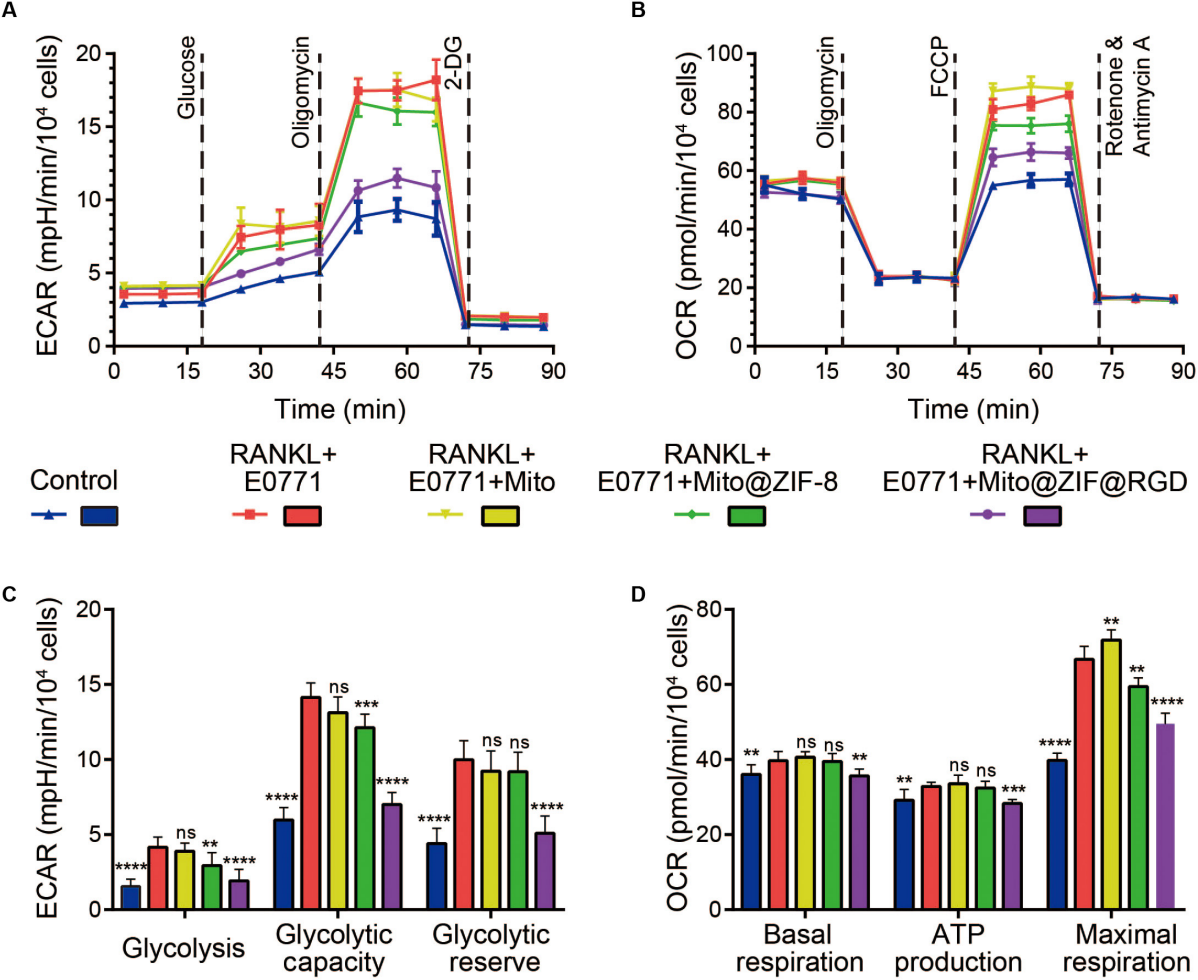
Metabolic remodeling of cancer-conditioned osteoclast precursor cells following each group treatment. (A) Seahorse XF analysis showing the time-course of extracellular acidification rate (ECAR) in osteoclast precursor cells across different treatment groups. (B) Seahorse XF analysis showing the time-course of oxygen consumption rate (OCR) in different treatment groups. (C) Glycolysis-related parameters derived from ECAR profiles, including basal glycolysis, glycolytic capacity, and glycolytic reserve, across different treatment groups. (D) Mitochondrial respiration parameters derived from OCR profiles, including basal respiration, adenosine triphosphate (ATP) production, and maximal respiration, across different treatment groups. *n* = 3, **P < 0.01, ***P < 0.001, ****P < 0.0001. ns, not significant.

Subsequently, we investigated the effects of Mito@ZIF@RGD on metabolic reprogramming in differentiating osteoclast precursor cells. ATP assays showed that, compared with the RANKL group and the RANKL + E0771 group, all treatment groups-including matched Zn^2+^, ZIF@RGD, Mito@ZIF-8, inactive Mito@ZIF@RGD, and Mito-RGD-tended to reduce ATP production to varying degrees. Notably, the Mito@ZIF@RGD group exhibited the most pronounced effect, with ATP levels approaching those observed under baseline-like metabolic conditions (Fig. S10). Furthermore, Seahorse XF analysis was performed to evaluate extracellular acidification rate (ECAR) and oxygen consumption rate (OCR), reflecting glycolytic activity and mitochondrial oxidative phosphorylation, respectively. The results indicated that both ECAR and OCR were markedly elevated in the RANKL + E0771 group and the RANKL + E0771 + Mito group, suggesting enhanced metabolic activity. Specifically, glycolysis-related parameters—including basal glycolysis, glycolytic capacity, and glycolytic reserve—were increased, indicating activation of glycolytic pathways (Fig. 7A and C). In parallel, OCR analysis showed elevated basal respiration, ATP production, and maximal respiration capacity, suggesting a concurrent up-regulation of oxidative phosphorylation (Fig. 7B and D). In contrast, treatment with Mito@ZIF-8 and Mito@ZIF@RGD reduced both ECAR and OCR levels. Notably, the Mito@ZIF@RGD group showed the strongest tendency toward suppression of glycolytic activation and partially normalized the abnormally elevated maximal respiratory capacity, with cellular metabolic profiles shifting closer to baseline levels (Fig. 7). Collectively, these findings suggest that costimulation with RANKL and tumor-derived signals may induce a coupled enhancement of glycolysis and oxidative phosphorylation in osteoclast precursor cells, whereas mitochondrial transplantation—particularly Mito@ZIF@RGD—may effectively reverse this metabolic reprogramming and contribute to the restoration of metabolic homeostasis.

### In vivo therapeutic efficacy of Mito@ZIF@RGD in breast cancer-induced bone osteolysis model

To evaluate the in vivo biosafety of Mito@ZIF@RGD, a mouse model with intra-articular administration was first established. Briefly, these nanoparticle platforms (Mito, Mito@ZIF-8, and Mito@ZIF@RGD) was injected into the knee joints of mice every 7 d, followed by normal feeding for 15 d. Subsequently, major organs including the heart, liver, spleen, lungs, and kidneys were harvested for hematoxylin and eosin (H&E) staining (Fig. S11). The histological results revealed no obvious pathological abnormalities, indicating that Mito@ZIF@RGD did not induce appreciable systemic toxicity in vivo. In addition, serum biochemical parameters, including alanine aminotransferase (ALT), aspartate aminotransferase (AST), blood urea nitrogen (BUN), and inflammatory cytokine levels, were evaluated 24 h after the first administration. The results showed no significant differences in ALT and AST levels among all groups (Fig. S12A and B), suggesting that the treatments did not induce detectable hepatic dysfunction. Similarly, no statistically significant changes in BUN levels were observed (Fig. S12C), indicating the absence of apparent renal impairment. Furthermore, the serum levels of proinflammatory cytokines, including interferon-γ, tumor necrosis factor-α, and interleukin-6, remained unchanged across all groups (Fig. S12D to F), suggesting that the treatment did not trigger systemic inflammatory responses or immune activation. Collectively, these results indicate that all treatment groups maintained stable hepatic and renal function parameters as well as inflammatory cytokine levels, suggesting good in vivo biocompatibility and systemic safety of the developed system.

**Fig. 8.**
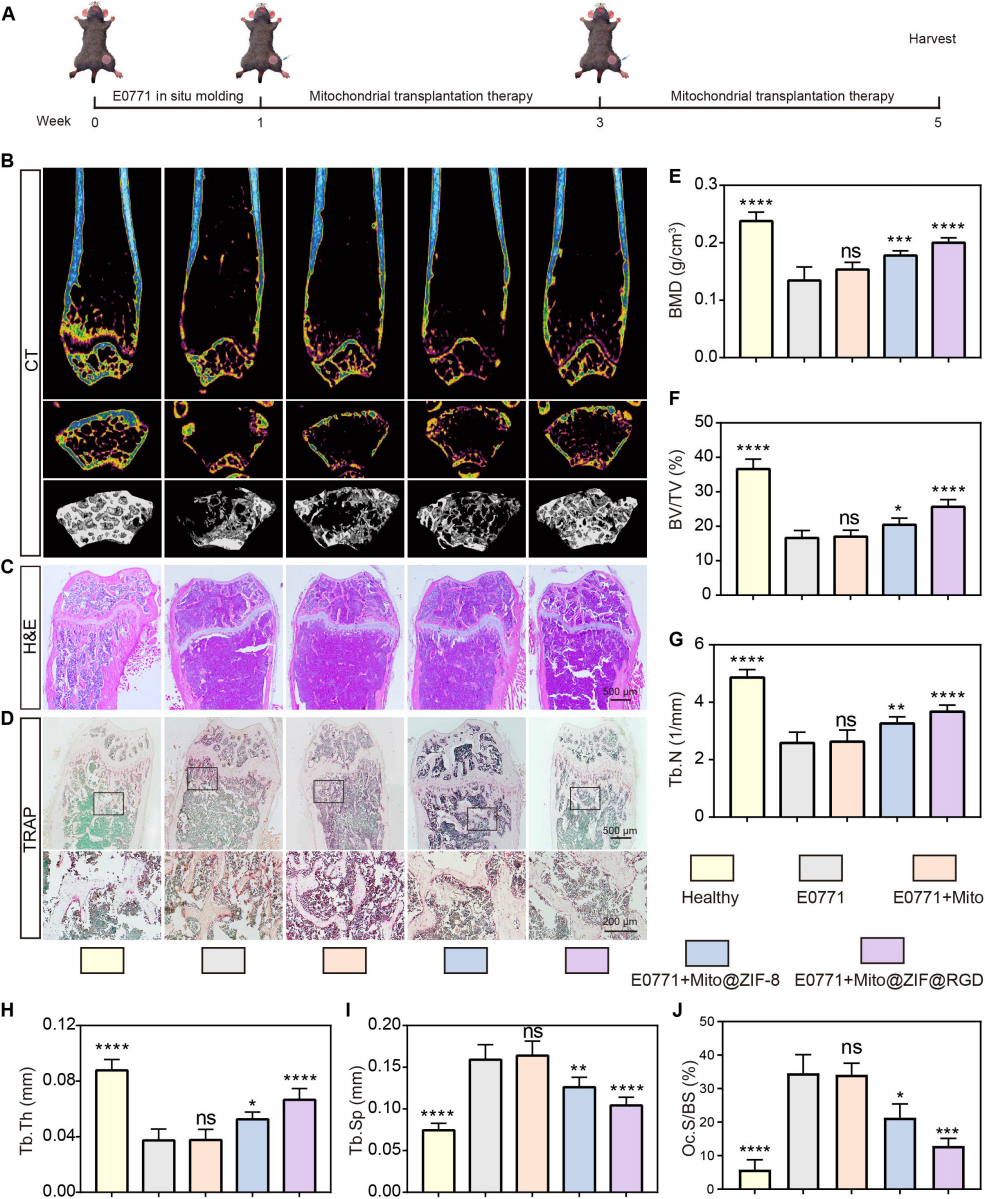
In vivo efficacy of Mito@ZIF@RGD in breast cancer-induced bone osteolysis. (A) Schematic illustration of the in vivo therapeutic procedure. (B) Representative 2-dimensional and 3-dimensional reconstructed micro-computed tomography (micro-CT) images. (C) Representative H&E staining images of femoral sections from the breast cancer-induced bone osteolysis model. (D) Representative tartrate-resistant acid phosphatase (TRAP) staining images of femoral sections from the breast cancer-induced bone osteolysis model. (E to I) Quantitative micro-CT analysis of bone-related parameters, including bone mineral density (BMD), bone volume fraction (BV/TV), trabecular number (Tb.N), trabecular thickness (Tb.Th), and trabecular separation (Tb.Sp). (J) Percentage of TRAP-positive osteoclast area in femoral sections. Each group: healthy mice (Healthy), E0771 tumor-bearing mice (E0771), E0771 tumor-bearing mice treated with mitochondria alone (E0771 + Mito), E0771 tumor-bearing mice treated with Mito@ZIF-8 (E0771 + Mito@ZIF-8), and E0771 tumor-bearing mice treated with Mito@ZIF@RGD (E0771 + Mito@ZIF@RGD). Scale bars represent 200 and 500 μm, respectively, *n* = 5, *P < 0.05, **P < 0.01, ***P < 0.001, ****P < 0.0001. ns, not significant.

Thereafter, an orthotopic E0771 breast cancer-associated bone osteolysis mouse model was established to assess therapeutic efficacy (Fig. 8A). Intra-articular injection of nanoparticles showed no obvious impact on tumor growth in tumor-bearing mice (Fig. S13A). Meanwhile, zinc ion levels in joint homogenates from nanoparticle-treated tumor-bearing mice were close to the physiological range observed in healthy mice (Fig. S13D) and remained far below the reported lethal toxicity threshold (2,000 mg/kg) [29]. Bone structural integrity and osteoclast activity were evaluated using micro-computed tomography (micro-CT), H&E staining, TRAP staining, and corresponding quantitative analyses. The results are presented in Fig. 8, including representative micro-CT images and histological assessments (Fig. 8B) demonstrated that compared with the healthy control group (Healthy), the E0771 model group and Mito group exhibited obvious bone destruction, characterized by sparse and fractured trabecular bone and markedly reduced bone volume. In contrast, the Mito@ZIF-8 group only showed slight improvement in bone destruction, while the Mito@ZIF@RGD group maintained relatively intact trabecular bone structure, with markedly inhibited bone destruction. H&E staining results (Fig. 8C) further confirmed that the E0771 group had disorganized bone tissue morphology and interrupted continuity of bone matrix, while the Mito@ZIF@RGD group displayed clear and continuous bone matrix structure, which was closer to the healthy control group. TRAP staining (Fig. 8D) showed a significant increase in the number of TRAP-positive osteoclasts on the bone surface in the E0771 group, whereas the Mito@ZIF@RGD group had a marked reduction in TRAP-positive cells, which were only sparsely distributed. For quantitative analysis, micro-CT-related parameters (Fig. 8E to I) indicated that key indicators such as bone volume fraction, trabecular thickness, and bone mineral density in the E0771 group were significantly lower than those in the healthy control group. No significant improvement was observed in the Mito group, while all micro-CT parameters in the Mito@ZIF@RGD group were markedly improved, showing statistically significant differences compared with the E0771 group, and some parameters were close to the level of the healthy control group. Quantitative analysis of TRAP-positive area (Fig. 8J) showed that the Oc.S/BS value in the E0771 group was more than 6 times that of the healthy control group. The Oc.S/BS value in the Mito@ZIF@RGD group was significantly reduced compared with the E0771 group, with a reduction of more than 60%, while the Mito group only showed slight reduction, which did not reach statistical significance. Meanwhile, serum biochemical analysis was performed to quantify C-terminal telopeptide (CTX) and procollagen type I N-terminal propeptide (PINP) levels in mice. The results showed that Mito@ZIF@RGD treatment significantly attenuated the enhanced bone turnover observed in tumor-bearing mice (Fig. S13B and C). Collectively, these in vivo results suggest that intra-articular administration of Mito@ZIF@RGD operates within a safe therapeutic window and may exert potential therapeutic effects against breast cancer-induced bone destruction. The treatment helps maintain bone structural integrity and is associated with a significant reduction in osteoclast activity, highlighting its potential applicability for the treatment of breast cancer-related osteolytic bone disease.

## Discussion and Conclusion

Cancer-associated bone osteolysis remains a major clinical challenge, largely driven by tumor-induced remodeling of the bone microenvironment [7]. This process places osteoclast precursors into a pathological “metabolic hotspot” state, characterized by excessive metabolic activation and uncontrolled osteoclast differentiation [8,30,31]. Current antiresorptive therapies, including bisphosphonates and RANKL inhibitors, primarily act by suppressing the activity of mature osteoclasts or blocking osteoclastogenic signaling pathways [32,33]. However, these approaches fail to correct the upstream metabolic imbalance that underlies osteoclast hyperactivation, resulting in a substantial unmet clinical need. To address this critical limitation, we developed a mitochondrial transplantation system (Mito@ZIF@RGD) and systematically validated its therapeutic efficacy through both in vitro and in vivo studies. By directly intervening at the metabolic origin of osteoclast dysregulation, this strategy establishes a novel prospect for treating cancer-induced bone osteolysis through metabolic reprogramming rather than conventional inhibition of osteoclast activity.

Mitochondrial transplantation, as an emerging strategy for cellular metabolic reprogramming, is currently limited by several critical challenges, including the instability of the in vivo microenvironment, the lack of targeting capability, and the difficulty in maintaining intrinsic mitochondrial functionality after transplantation [34,35]. In conventional mitochondrial transplantation approaches, naked mitochondria are highly susceptible to degradation by endogenous enzymes, oxidative stress, and phagocytic clearance, leading to a rapid loss of mitochondrial function, particularly ATP-producing capacity, and preventing effective delivery to disease sites for metabolic repair [34,36]. In this study, we developed a Mito@ZIF@RGD transplantation system through a biomimetic in situ mineralization strategy on mitochondria. The hierarchical coordination of ZIF-8 on the surface of healthy mitochondria forms a nanocrystalline shell that serves as a protective barrier, preserving mitochondrial integrity and function. By constructing this functional protective coating, our approach fundamentally addresses the long-standing challenge of functional instability in mitochondrial transplantation (Fig. S1). Our results demonstrate that the ZIF-8 shell plays a critical role in maintaining mitochondrial functionality during the transplantation process. In vitro assessments showed that ZIF-8 encapsulation sustained mitochondrial ATP production over 48 h, far exceeding the rapid activity decline of bare mitochondria (Fig. 2A, B, and H). This protective effect translated to in vivo efficacy: ZIF-8-encapsulated mitochondria (Mito@ZIF-8) partially mitigated bone destruction compared to the breast cancer-induced bone osteolysis model, while bare mitochondria showed no significant improvement (Fig. 8). The pH-responsive degradation of ZIF-8, triggered by the acidic microenvironment of tumor-acclimated bone marrow osteoclast precursor cells (pH < 6.8), enabled mitochondria were released specifically at differentiating osteoclast precursor cells, avoiding premature inactivation in neutral systemic circulation. This pH-dependent release behavior was successfully recapitulated in vitro (Fig. S2). Complementing this, RGD-mediated active targeting to integrin αvβ3 substantially enhanced delivery efficiency. In vitro, under tumor cell coculture conditions, integrin αVβ3 expression is progressively up-regulated during the early stage of osteoclast differentiation (Fig. S3). In addition, RGD modification increased the phagocytosis rate of differentiating osteoclast precursor cells by nearly 50% compared to nontargeted Mito@ZIF-8 (Fig. 4); the enhanced delivery efficiency was closely associated with the up-regulated expression of αVβ3 in differentiating osteoclast precursor cells (Figs. S3 and S4). Also, in vivo, this translated to superior bone protection (Fig. 8D), with trabecular bone volume fraction and mineral density restored to near-healthy levels (Fig. 8E and F). Notably, histological assessments revealed no tissue inflammation or toxicity in Mito@ZIF@RGD-treated animals (Figs. S11 and S12), consistent with in vitro cytocompatibility data (Fig. 3) showing no cytotoxicity even after long-term coculture, confirming the system’s balance of efficacy and safety.

Extensive studies have demonstrated that cancer-induced osteolysis is driven by RANKL, lactate, and inflammatory mediators released from the tumor-conditioned bone marrow microenvironment [5,6]. These pathological stimuli disrupt ionic homeostasis in osteoclast precursor cells, particularly zinc ion balance, which is essential for maintaining mitochondrial functional integrity and redox stability [13–16]. Under tumor-associated inflammatory conditions, sustained efflux and depletion of intracellular Zn^2+^ impair the activity of multiple zinc-dependent mitochondrial enzymes, destabilize the mitochondrial membrane potential, and promote electron leakage from the respiratory chain [37,38]. This cascade results in excessive accumulation of ROS, thereby amplifying osteoclastogenic signaling and accelerating osteolytic progression [15,16]. In this study, treatment with Mito@ZIF@RGD not only enabled efficient protection and targeted delivery of healthy mitochondria but also served as a zinc ion reservoir with sustained release capability through its ZIF-8 shell. Our results demonstrated that Mito@ZIF@RGD underwent gradual degradation and cargo release within tumor-conditioned osteoclast precursor cells, a process further validated by in vitro pH-responsive release experiments (Fig. S2). Importantly, fluorescence imaging revealed that Mito@ZIF@RGD treatment effectively restored intracellular zinc ion levels to a state comparable to that observed prior to tumor conditioning (Fig. 5B and C). Mito@ZIF@RGD treatment was associated with improved mitochondrial membrane potential in breast cancer-induced osteoclast precursor cells (Fig. S6), which may contribute to the regulation of ROS production during osteoclast differentiation (Fig. 6B and Fig. S7). Consistent with zinc homeostasis recovery, Western blot analyses showed that stabilization of intracellular Zn^2+^ levels has the potential ability to preserve mitochondrial membrane-associated respiratory complexes I and III (Fig. 5D). The stabilization of these complexes is critical for preventing mitochondrial electron leakage and subsequent ROS amplification. In parallel, the recovery of Zn^2+^-dependent antioxidant enzymes, including SOD1 and GSR (Fig. 5D), may support intracellular superoxide anion clearance and glutathione regeneration, contributing to improved redox homeostasis [39–41]. Moreover, OCR/ECAR analysis (Fig. 7) further suggested that Mito@ZIF@RGD treatment helped rebalance cellular energy metabolism by alleviating the excessive activation of mitochondrial respiration and glycolytic flux in tumor-acclimatized osteoclast precursor cells. This metabolic normalization may reduce the burden on electron transport and glycolytic stress, thereby contributing to decreased ROS accumulation and the maintenance of mitochondrial function. Collectively, these synergistic actions may establish a dual protective mechanism that maintains mitochondrial integrity and effectively suppresses tumor acclimatization-induced oxidative stress after mitochondrial transplantation.

As described above, dysregulated differentiation of tumor-conditioned osteoclast precursor cells is driven by mitochondria-centered metabolic dysfunction, which cannot be effectively corrected by conventional therapies that primarily target downstream pathways [5,7,8]. In contrast, the Mito@ZIF@RGD mitochondrial transplantation platform developed in this study appears to support upstream metabolic regulation by enhancing mitochondrial delivery efficiency and improving intracellular zinc ion homeostasis in recipient cells, which may contribute to mitochondrial metabolic repair. In vitro experiments demonstrated that Mito@ZIF@RGD directly reversed intracellular ROS accumulation, reducing ROS levels by approximately 77% (Fig. 6B and F and Fig. S7), and markedly down-regulated the phosphorylation or expression of p-p65, p-ERK, c-Fos, and NFATc-1 (Fig. 6E and H to K), ultimately suppressing osteoclast differentiation by more than 85% (Fig. 6C, D, and G and Figs. S8 and S9). In vivo, this mechanism translated into substantial skeletal protection. Quantitative analyses revealed that, compared with the disease model group, Mito@ZIF@RGD treatment reduced TRAP-positive osteoclast surface (Oc.S/BS) by more than 55% (Fig. 8D and J), accompanied by pronounced improvements in bone microarchitecture, structural parameters, and bone turnover efficiency (Fig. 8B, C, and E to I and Fig. S13B and C). Importantly, unlike nonspecific mitochondrial inhibitors such as rotenone [42], Mito@ZIF@RGD may help regulate metabolic homeostasis without causing broad suppression of mitochondrial function. This fundamental distinction is reflected in the preservation of cellular proliferation in vitro and the recovery of bone structure in vivo. Histological analyses further showed sustained integrity of the bone matrix approaching that of healthy controls. Together, these results suggest that Mito@ZIF@RGD-mediated metabolic modulation may contribute to the attenuation of pathological osteolysis and support a microenvironment more conducive to bone repair.

Compared to current treatments, Mito@ZIF@RGD offers 3 key advantages. First, RGD modification improved mitochondrial delivery through interaction with integrin αvβ3, potentially reducing nonspecific cellular uptake compared with nontargeted approaches (which affect healthy osteoclasts and increase risks like osteonecrosis of the jaw), as confirmed by in vivo observations of reduced osteoclast activity without disrupting other bone cell populations. Second, it exerts dual effects: While RANKL inhibitors only reduce osteoclast numbers without repairing bone damage, Mito@ZIF@RGD both inhibits bone resorption and restores trabecular volume and mineral density, suggesting potential anabolic activity that warrants further investigation (e.g., assessing osteoblast markers like alkaline phosphatase). Third, the ZIF-8 shell enables sustained efficacy: A single administration zinc ion release provides long-term functional maintenance after mitochondrial transplantation to protect bones, unlike bisphosphonates and RANKL inhibitors which require repeated dosing, potentially improving patient compliance. These advantages position Mito@ZIF@RGD as a promising clinical candidate, with future potential for combination with existing therapies (e.g., denosumab) to enhance efficacy or pharmacokinetic optimization (e.g., PEGylation for prolonged circulation) to improve bioavailability.

Despite promising results, this study has limitations that need addressing for clinical translation. First, in vivo experiments used a single breast cancer cell line in an orthotopic model: Breast cancer heterogeneity (e.g., triple-negative subtypes with higher bone metastasis rates) requires validation in multiple subtypes and patient-derived xenograft models to reflect clinical diversity. Second, long-term safety and biodegradation of ZIF-8 need evaluation: While short-term data show no toxicity, 3- to 6-month studies are needed to assess metabolism and excretion of degradation products (e.g., zinc ions) and avoid systemic accumulation risks (e.g., hepatotoxicity). Third, the optimal administration route was not explored: Local delivery (e.g., intraosseous injection) may enhance mitochondrial accumulation in localized bone lesions, and future pharmacokinetic studies should compare different routes (veins, joint cavities, and tumor sites, etc.) to maximize efficacy while minimizing systemic exposure, and further studies will be conducted on the distribution characteristics, targeting efficiency, and safety after administration. Fourth, the present study primarily delineates the mechanisms by which biomineralization enhances mitochondrial delivery, along with its synergistic effects on mitochondrial function recovery and Zn^2+^ homeostasis, at the functional and phenotypic levels. Nevertheless, the underlying molecular causality remains to be fully elucidated. Future studies will integrate metabolic flux analysis, including stable isotope tracing, with targeted interrogation of key regulatory pathways to further define the causal interplay between mitochondrial functional remodeling and Zn^2+^ homeostasis. Finally, effects on breast cancer cells themselves were not investigated: Confirming no promotion of cancer proliferation, migration, or metastasis is critical for clinical utility, requiring future assessments in coculture and in vivo metastasis assays.

In conclusion, we developed a Mito@ZIF@RGD nanotransplantation platform in which RGD modification improved the delivery efficiency of healthy mitochondria to differentiating osteoclast precursor cells. Within cells, Mito@ZIF@RGD responds to the acidic microenvironment to release mitochondria and Zn^2+^, thereby facilitating mitochondrial transplantation while partially correcting cancer-induced zinc ion imbalance in differentiating osteoclast precursor cells. This coordinated mechanism may help support posttransplant mitochondrial functionality and attenuate cancer-associated bone loss in vivo. By integrating structural stability, enhanced cellular delivery, and metabolic support into a single mitochondrial transplantation system, this work provides a potentially effective strategy for mitochondrial delivery to differentiating osteoclast precursor cells. Collectively, this platform offers a promising therapeutic approach for cancer-associated bone loss and provides a basis for further development of mitochondrial transplantation-based metabolic therapies.

## Materials and Methods

### Animal model establishment

All animal experiments were conducted in strict accordance with the National Institutes of Health Guide for the Care and Use of Laboratory Animals and relevant regulations on laboratory animal management in China. All procedures were approved by the Ethics Committee of Shanghai Shengchang Biotechnology Co., Ltd. (Approval No. 2023-08-SGKYJS-CWG-050). Six- to 8-week-old healthy female C57BL/6 mice (specific pathogen-free grade) were used and maintained under standard specific pathogen-free conditions (temperature 22 ± 2 °C, relative humidity 50% to 60%, 12-h light/dark cycle) with ad libitum access to food and water. Animals were acclimatized for 7 d prior to experimentation. A breast cancer bone microenvironment model was established by resuspending exponentially growing E0771 cells to a concentration of 5 × 10^4^ cells per 100 μl PBS, followed by orthotopic injection into the bilateral fourth mammary fat pads of mice under sterile conditions. Tumor formation was monitored regularly, and successful model establishment was confirmed approximately 7 d postinoculation, after which animals were randomly assigned to different experimental groups. Randomization was performed using a random number table. Each group contained *n* = 5 mice, and group allocation was carried out by an independent operator to minimize experimental bias. After model establishment, 100 μl of different nanoparticle formulations (1:500 dilution stock solution) was administered via intra-articular injection into the knee joint. Injections were performed once every 3 d for 3 consecutive weeks. All experimental conditions were kept consistent across groups, with the nanoparticle formulation as the only variable. At the end point, mice were euthanized, and bilateral femurs and joint tissues were harvested for micro-CT analysis, histological staining (H&E and TRAP), and molecular biological assays. In parallel, serum and synovial joint fluids were collected for the analysis of CTX, PINP, and Zn^2+^ levels. Animals that failed to meet tumor model criteria or died from nonexperimental causes were excluded from final statistical analyses.

### Cell culture

E0771 mouse breast cancer cells and RAW264.7 cells were purchased from the American Type Culture Collection. As previously described [43–45], both cell lines were cultured in Dulbecco’s modified Eagle medium (Thermo Fisher Scientific, USA). Isolation of mouse BMDMs was performed as previously reported. Under sterile conditions, bone marrow cells were flushed from C57BL/6 mice and treated with red blood cell lysis buffer (Beyotime, CN) to remove erythrocytes. The resulting cells were then plated in culture dishes and incubated at 37 °C for 14 h. The supernatant was collected and supplemented with 30 ng/ml macrophage colony-stimulating factor (M-CSF, Bio-Techne Corporation, USA), followed by adherent culture for 3 d to obtain BMDMs. Dulbecco’s modified Eagle medium was supplemented with 10% fetal bovine serum (Gibco, Thermo Fisher Scientific, USA) and 1% antibiotics (100 U·ml^−1^ penicillin and 100 μg·ml^−1^ streptomycin, Thermo Fisher Scientific, USA) for the culture of all aforementioned cells. Cells were maintained in an incubator at 37 °C with 5% CO₂, and the medium was changed every other day. Bone marrow mesenchymal stem cells were isolated from rat femurs and tibias. After both ends of the bones were cut, the bone marrow cavity was repeatedly flushed with α-minimum essential medium supplemented with 10% fetal bovine serum and 1% penicillin–streptomycin to collect the bone marrow cell suspension. The cell suspension was filtered through a 70-μm cell strainer and centrifuged. After discarding the supernatant, the cell pellet was resuspended in complete α-minimum essential medium and seeded for culture. After 24 to 48 h, the medium was replaced to remove nonadherent cells, and the medium was subsequently changed every 2 to 3 d. When the cells reached 80% to 90% confluence, they were passaged using 0.25% trypsin–EDTA.

### Mitochondrial extraction and mineralization

Mitochondrial extraction and mineralization were performed according to previously established protocols. Briefly, cells were cultured to 90% confluence. For mitochondrial fluorescence labeling experiments, cells were incubated with 50 nM MitoTracker Red CMXRos (Thermo Fisher Scientific, USA) overnight, washed with PBS, and harvested. Mitochondria were isolated under ice bath conditions at 4 °C using a Mitochondrial Isolation Kit (Thermo Fisher Scientific, USA). The protein concentration of the extracted mitochondria was determined by the bicinchoninic acid (BCA) assay. The isolated mitochondria were resuspended in 70 μl of pre-prepared 40 mM zinc acetate solution (Sigma-Aldrich, USA), followed by dropwise addition of 70 μl of 160 mM 2-methylimidazole solution (Sigma-Aldrich, USA). Mineralization was carried out under gentle stirring with a magnetic stirrer for 3 min. Mineralized mitochondria (Mito@ZIF-8) were collected by low-speed centrifugation and resuspended in 0.9% sodium chloride solution for further use. To achieve preosteoclast targeting, 1 mg/ml cRGD peptide (cRGDfK, MCE, USA) or RGE (Arg-Gly-Glu, ChinaPeptides Co., Ltd.,CN) prepared in 0.9% sodium chloride solution was added to the centrifuged Mito@ZIF-8, followed by gentle stirring for 10 min. Unbound cRGD or RGE was removed by centrifugation, and the mixture was resuspended in 200 μl of 0.9% sodium chloride solution to prepare a stock solution for later use, thus preparing mineralized mitochondria (Mito@ZIF@RGD) that target osteoclast precursors.

### TEM

Six microliters of the sample was dropped onto copper grids and allowed to stand overnight to evaporate excess liquid. Images were then acquired using a transmission electron microscope (JEM-1400, Japan). For observation of sectioned samples, the prepared samples were fixed with 2.5% glutaraldehyde for 2 h and 1% osmium tetroxide for 1 h, respectively. Samples were dehydrated through a graded ethanol series, embedded in resin (MCE, USA), and sectioned into 70-nm-thick slices using an ultramicrotome (Leica UC7, Germany). Sections were stained with 2% uranyl acetate and 0.5% lead citrate, and images were captured under a TEM at 100 kV.

### Coomassie Brilliant Blue staining

10% sodium dodecyl sulfate-polyacrylamide gel electrophoresis gels were prepared. Mitochondrial protein solutions were obtained by lysing samples with radioimmunoprecipitation assay lysis buffer and boiling for subsequent use. Thirty micrograms of protein solution was subjected to electrophoresis at 120 V for 40 min. The gel was then stained with Coomassie Brilliant Blue R-250 solution (Thermo Fisher Scientific, USA) for 30 min, followed by washing with deionized water to remove excess stain until clear bands appeared. Finally, images were acquired using a Bio-Rad ChemiDoc MP imaging system.

### Dynamic light scattering and zeta potential measurement

Synthesized mitochondria and their mineralized samples were resuspended in 0.9% sodium chloride solution to a final concentration of 0.5 mg/ml. Samples were added to a cuvette, and particle size and zeta potential parameters were measured using a Zetasizer Nano instrument (Malvern Instruments, UK).

### Zn^2+^ release detection from Mito@ZIF@RGD under different pH conditions

The pH-responsive Zn^2+^ release behavior of Mito@ZIF@RGD was evaluated under different conditions. Briefly, 1 ml of Mito@ZIF@RGD nanoparticle suspension prepared by the biomineralization method was incubated in buffer solutions at pH 6.5 or pH 7.4 at 37 °C with gentle shaking. At predetermined time points (0, 2, 4, 8, 16, and 24 h), the samples were centrifuged at 3,500 rpm for 3 min, and 50 μl of the supernatant was collected and replaced with an equal volume of fresh buffer. The collected supernatants were diluted 1,000-fold, and Zn^2+^ concentrations were quantified using a zinc ion assay kit (Beyotime, S1077S, CN) according to the manufacturer’s instructions. Absorbance was measured at 560 nm using a microplate reader. Zn^2+^ concentrations at each time point was calculated based on a standard calibration curve, and cumulative Zn^2+^ release profiles were subsequently determined.

### ATP assay

ATP concentration was determined according to previously described methods. As previously described [46], the protein concentration of the extracted mitochondria or cell lysate was determined using the BCA method, and the mitochondrial or cell protein concentration of each group was adjusted to be consistent. Diluted ATP standard curve reaction solution, ATP standard solution, and sample test solution were added to a microtiter plate according to the instructions of the ATP Assay Kit (Thermo Fisher Scientific, USA). After adding the test reaction solution, the total volume was adjusted to 100 μl, with 3 replicates per group. Following incubation at room temperature for 15 min, luminescence intensity was detected using a Varioskan FLASH microplate reader (Thermo Fisher Scientific, USA). ATP production of each sample was calculated using the standard curve.

### Live/dead assay

As previously described [47,48], 5 × 10^4^ RAW264.7 cells per well were seeded in 24-well plates with 3 replicates per group and cultured 24 h in 50 ng/ml RANKL. Mitochondrial samples from each group were then added to the wells and cultured for 1, 3, and 5 d. Cells were gently washed with PBS to remove residual nanoparticles. Two hundred microliters of Live/Dead working solution (2 μM Calcein-AM, 4 μM PI, Beyotime, CN) was added to each well, and cells were incubated in the dark at 37 °C for 20 min. Images were captured using a fluorescence microscope.

### CCK-8 assay

A total of 5 × 10^4^ RAW264.7 cells per well were seeded in 24-well plates with 3 replicates per group and cultured 24 h in 50 ng/ml RANKL. Mitochondrial samples from each group were then added to the wells and cultured for 1, 2, and 3 d. Cells were gently washed with PBS to remove residual nanoparticles. One hundred microliters of CCK-8 working solution (Beyotime, CN) was added to each well, and cells were incubated in the dark at 37 °C for 2 h. Absorbance was measured at 450 nm using a microplate reader.

### Seahorse metabolic flux analysis (OCR/ECAR)

To investigate metabolic reprogramming of osteoclast precursor cells, we evaluated mitochondrial respiration and glycolytic activity using a Seahorse XF analyzer (Agilent Technologies, Inc.). RAW264.7 cells cocultured with E0771 cells were treated with different nanoparticles for 24 h. The OCR was measured using the Seahorse XF mitochondrial stress assay after sequential administration of oligomycin, carbonyl cyanide 4-(trifluoromethoxy)phenylhydrazone, and rotenone/antimycin A. The ECAR was measured using the Seahorse XF glycolytic stress assay after sequential addition of glucose, oligomycin, and 2-deoxy-D-glucose. Key metabolic parameters, including basal respiration, maximal respiration, ATP-coupled respiration, basal glycolysis, and glycolytic capacity, were calculated.

### Flow cytometry

As previously described [49,50], 2 × 10^5^ RAW264.7 cells per well were seeded in 6-well plates with 3 replicates per group and cultured 24 h in 50 ng/ml RANKL. MitoTracker Red CMXRos-labeled mitochondrial samples from each group were added to the wells and cultured for 24 h. Cells were collected, and the ability of RAW264.7 cells to phagocytose mitochondria from each group was detected using a flow cytometer (BD, USA).

### CLSM

A total of 3 × 10^4^ RAW264.7 cells per well were seeded in confocal dishes with 3 replicates per group and cultured 24 h in 50 ng/ml RANKL. Recipient cell mitochondria were labeled with MitoTracker Green FM (Thermo Fisher Scientific, USA), whereas donor mitochondria were labeled with MitoTracker Red CMXRos (Thermo Fisher Scientific, USA). MitoTracker Red CMXRos-labeled mitochondrial samples from each nanoparticle group were added to the dishes and cultured for 24 h. Cells were then fixed and permeabilized. F-actin was stained with Actin-Tracker Green 488 (Beyotime, CN) for 1 h, followed by staining with 4′,6-diamidino-2-phenylindole (Yuanye, CN) for 15 min. Samples were mounted with antifluorescence quenching medium, and images were acquired using a confocal laser scanning microscope (Carl Zeiss, Germany).

### Intracellular Zn^2+^ detection

RAW264.7 cells (8 × 10^4^) were seeded in the lower chamber of a 24-well transwell coculture system, while E0771 cells (4 × 10^4^) were seeded in the upper chamber and cocultured 24 h in 50 ng/ml RANKL. Subsequently, mitochondrial samples from different groups were added to the RAW264.7 cells in the lower chamber and incubated for 24 h. After treatment, the upper chamber cells and culture medium were removed, and the RAW264.7 cells were washed 3 times with PBS. FluoZin-3 AM (Thermo Fisher Scientific, USA) was prepared as a working solution (2 μM) according to the manufacturer’s instructions, and 0.5 ml was added to each well followed by incubation at 37 °C for 30 min in the dark. Intracellular Zn^2+^ levels were quantified by FCM using the fluorescein isothiocyanate channel. In addition, cytoskeletal staining (Thermo Fisher Scientific, USA) and nuclear staining (YuanYe, CN) were performed as described previously, and fluorescence images were acquired using a confocal laser scanning microscope.

### ROS assay

A total of 5 × 10^4^ RAW264.7 cells were seeded in the lower chamber of 24-well transwell plates, and 2 × 10^4^ cells were seeded in the upper chamber. Cells were cocultured 24 h in 50 ng/ml RANKL. Mitochondrial samples from each group were added to the lower chamber for 24 h of intervention. The upper chamber cells and lower chamber culture medium were discarded, and cells were washed 3 times with PBS. Cells in the lower chamber were stained with 10 μM 2′,7′-dichlorodihydrofluorescein diacetate probe according to the manufacturer’s instructions and incubated at 37 °C for 25 min. After washing 3 times with PBS, ROS images were captured using a fluorescence microscope.

### JC-1 staning

After treatment with different nanoparticle formulations for 24 h, the mitochondrial membrane potential (ΔΨm) of RAW264.7 cells RAW264.7 cells cocultured with E0771 cells was evaluated using the JC-1 Mitochondrial Membrane Potential Assay Kit (Beyotime Biotechnology Co., Ltd., CN) according to the manufacturer’s instructions. Briefly, cells were washed with PBS and incubated with JC-1 staining working solution at 37 °C for 20 min in the dark. After staining, cells were washed twice with JC-1 staining buffer to remove excess dye. Fluorescence images were subsequently captured using a fluorescence microscope.

### Cellular TRAP staining

As previously described [51], BMDMs were seeded in the lower chamber of transwell plates, and E0771 cells were seeded in the upper chamber. After overnight coculture, mitochondrial samples from each group were added to the lower chamber for 7 d of intervention with 30 ng/ml M-CSF and 50 ng/ml RANKL. As previously described, TRAP staining was performed using a TRAP Staining Kit (Sigma, USA) to observe osteoclast fusion. Briefly, TRAP staining working solution was prepared in preheated deionized water according to the kit instructions, and cells were incubated in the dark at 37 °C for 45 min. After washing off the staining solution, TRAP-stained images were acquired using an inverted microscope. TRAP-positive multinucleated cells containing ≥5 nuclei were defined as mature osteoclasts. The TRAP-positive area ratio was calculated as the percentage of TRAP-positive osteoclast area relative to the entire field of view. Quantification was performed using ImageJ software, with 3 independent replicates per group. Statistical comparisons were performed using 1-way analysis of variance (ANOVA) followed by Tukey’s post hoc test, and *P* < 0.05 was considered statistically significant.

### Bone resorption assay

BMDMs were seeded onto Osteo Assay Plates (Corning, USA), and E0771 cell culture supernatant was used as the conditioned medium. After overnight culture, mitochondrial samples from each group were added to the Osteo Assay Plates and incubated for 7 d in the presence of 30 ng/ml M-CSF and 50 ng/ml RANKL. Following osteoclast differentiation and bone resorption induction, the plates were treated with 5% sodium hypochlorite for 10 min to remove adherent cells. After 3 washes with deionized water, the remaining resorption pits were visualized using a bright-field microscope. Representative resorption areas were outlined with red dashed lines in the images. Quantification of the resorption areas was performed using ImageJ software, the percentage of bone resorption area was calculated as the ratio of the resorbed area to the total analyzed area. Three independent replicates analyzed for each group. Statistical analysis was conducted using 1-way ANOVA followed by Tukey’s post hoc test, and *P* < 0.05 was considered statistically significant.

### Lysosomal escape assay

RANKL-induced RAW264.7 cells were cocultured with MitoTracker Green-labeled Mito@ZIF@RGD for 0.5 and 4 h. MitoTracker Green FM (Thermo Fisher Scientific, USA) was used to label exogenous mitochondria. Subsequently, cells were stained with LysoTracker Red (Beyotime Biotech, CN) for lysosomal labeling and Hoechst (Beyotime Biotech, CN) for nuclear staining. The intracellular distribution and colocalization of exogenous mitochondria and lysosomes were visualized using CLSM to evaluate the intracellular trafficking and lysosomal escape capability of Mito@ZIF@RGD-delivered mitochondria.

### Reverse transcription quantitative polymerase chain reaction analysis

Total RNA was extracted from treated cells using TRIzol reagent, followed by chloroform and isopropanol (Beyotime, China) according to the manufacturer’s instructions. The concentration of RNA was measured using a NanoDrop spectrophotometer (Thermo Fisher Scientific, USA). Subsequently, 1 μg of total RNA from each sample was reverse-transcribed into complementary DNA using a reverse transcription kit (Takara, Japan). Quantitative real-time polymerase chain reaction was performed using gene-specific primers (listed in Table S1). Glyceraldehyde 3-phosphate dehydrogenase was used as an internal reference, and relative gene expression levels were calculated using the 2^−ΔΔCt^ method.

### Western blot analysis

RAW264.7 cells and BMDMs were seeded in the lower chamber of transwell plates, and E0771 cells were seeded in the upper chamber. After overnight coculture, mitochondrial samples from each group were added to the lower chamber for 1, 2, 3 or 5 d of intervention with 30 ng/ml M-CSF or/and 50 ng/ml RANKL. Western blot analysis was performed as previously reported [52]. Cells were lysed with radioimmunoprecipitation assay lysis buffer (Beyotime, CN), and supernatants were collected by high-speed centrifugation. Protein concentration was determined by the BCA assay (Beyotime, CN), and the protein concentration of each group was adjusted to be consistent. Thirty micrograms of protein was subjected to electrophoresis on 10% sodium dodecyl sulfate-polyacrylamide gel electrophoresis gels (XinSaimai, CN) and transferred to polyvinylidene fluoride membranes at a constant current of 300 mA for 60 min. Membranes were blocked with 5% nonfat milk solution for 1 h, followed by incubation with primary antibodies against p65 (ab32536, Abcam), phosphorylated (p)-p65 (sc-135769, Santa Cruz), ERK1/2 (AF0155, Affinity Biosciences), p-ERK1/2 (AF1015, Affinity Biosciences), c-Fos (AF0132, Affinity Biosciences), NFATc-1 (DF6446, Affinity Biosciences), UQCRC2 (99258T, CST), NDUFS1 (60153S, CST), SOD1 (37385T, CST), GSR (ab124995, Abcam), Integrin αV (ab179475, Abcam), Integrin β3 (ab119992, Abcam), and β-actin (58169s, CST) overnight at 4 °C. Membranes were washed 3 times with tris-buffered saline containing Tween 20 and incubated with corresponding secondary antibodies at room temperature for 1 h. After 3 additional washes with tris-buffered saline containing Tween 20, bands were incubated with enhanced chemiluminescence chemiluminescent substrate (Thermo Fisher Scientific, USA) and imaged using a Bio-Rad imaging system. β-Actin was used as an internal reference, and gray value analysis was performed using ImageJ software.

### CT

After sampling, mouse femur samples were fixed in 4% paraformaldehyde and subsequently scanned using a micro-CT scanner (Skyscan 1176, Bruker, Belgium). Five mouse femur samples from each group were included for quantitative micro-CT analysis (*n* = 5). The scanning parameters were set as follows: voltage, 70 kV; current, 200 μA; and spatial resolution, 9 μm per voxel. After scanning, the raw projection images were reconstructed using NRecon software, and consistent reconstruction parameters were applied to all samples to ensure comparability among groups. Trabecular bone microarchitecture in the distal femur was analyzed using CTAn software. The region of interest was defined within the trabecular bone area proximal to the distal femoral growth plate. To avoid interference from the growth plate, the region of interest started approximately 0.5 mm proximal to the growth plate and extended proximally to approximately 1.4 mm, corresponding to an analyzed region of approximately 0.9 mm in height. During analysis, cortical bone was manually excluded, and only the trabecular bone region was retained for 3-dimensional morphometric analysis. A unified grayscale threshold range (65 to 255, CTAn gray scale value) was applied to all samples for bone segmentation and binarization to ensure consistency across groups. Bone morphometric parameters, including bone mineral density, bone volume/total volume, trabecular thickness, trabecular number, and trabecular separation, were subsequently calculated.

### H&E staining

Bone tissue samples were fixed in 4% paraformaldehyde for 24 h, dehydrated, embedded in paraffin, and sectioned into 5-μm-thick slices. Staining was performed according to previously described protocols [52]. Briefly, samples were dewaxed twice with xylene, hydrated through a graded ethanol series, stained with hematoxylin for 3 min, differentiated with PBS, and then stained with eosin for 2 min. Excess stain was washed off with running water. Samples were dehydrated through a graded ethanol series, cleared twice with xylene, mounted with neutral balsam, and imaged using an inverted microscope.

### Bone tissue TRAP staining

Bone tissue samples were fixed in 4% paraformaldehyde for 24 h, dehydrated, embedded in paraffin, and sectioned into 5-μm-thick slices. Staining was performed according to previously described protocols [53]. Briefly, samples were dewaxed twice with xylene, hydrated through a graded ethanol series, and stained for osteoclasts in bone tissue using a TRAP Staining Kit (refer to cellular staining protocol). After washing off the TRAP stain with distilled water, samples were incubated with 1% methyl green solution (Beyotime, CN) for 2 min. Excess stain was washed off, and samples were dehydrated through a graded ethanol series, cleared twice with xylene, and mounted with neutral balsam. After drying, images were acquired using an inverted microscope, and the percentage of positive cells was analyzed using ImageJ software.

### Serum biochemical and bone metabolism analysis

At the end of the experiment, mice were anesthetized and blood samples were collected. After allowing coagulation at room temperature, serum samples were obtained by centrifugation. Commercial assay kits were used to measure serum levels of ALT, AST, BUN, interferon-γ, tumor necrosis factor-α, interleukin-6, C-terminal telopeptide of type I collagen (CTX-I), and PINP. All assays were performed according to the manufacturers’ instructions, and absorbance values were measured using a microplate reader. The concentrations of each parameter were calculated based on the corresponding standard curves and used to evaluate hepatic and renal function, inflammatory responses, and bone turnover following nanoparticle treatment.

### Statistical analysis

All experiments were performed with replicates (*n* = 3 or 5), and results are expressed as the mean ± SD. Graphs and statistical analyses were performed using Origin 8 and GraphPad Prism 8.0 software. Comparisons between samples were analyzed by 1-way analysis of variance (1-way ANOVA followed by Tukey’s post hoc test), and significant differences were indicated as *P* < 0.05, *P* < 0.01, *P* < 0.001, and *P* < 0.0001 (*P* < 0.05 is considered statistically significant). Quantitative analysis of image gray values or areas was performed using ImageJ software.

## Ethical Approval

All animal experiments were conducted in strict accordance with the National Institutes of Health Guide for the Care and Use of Laboratory Animals and relevant regulations on laboratory animal management in China. All procedures were approved by the Ethics Committee of Shanghai Shengchang Biotechnology Co., Ltd. (Approval No. 2023-08-SGKYJS-CWG-050).

## Data Availability

The data that support the findings of this study are available from the corresponding author upon reasonable request.
