# Supplementary material for: In Situ Biomineralization Enhances Mitochondrial Transplantation to Differentiating Osteoclast Precursors for Suppressing Cancer-Induced Osteolysis
Source: Research (Wash D C). 2026 Aug 19;9:1410. doi: 10.34133/research.1410 (PMC13486730; doi:10.34133/research.1410)
Supplement: Supplementary file 1 [file research.1410.f1.docx]

**Supporting Information**

**Title**

In Situ Biomineralization Enhances Mitochondrial Transplantation to Differentiating Osteoclast Precursors for Suppressing Cancer-Induced Osteolysis

Authors

Yu Zhang^1,2†^, Changpeng Liu^3†^, Pengzhen Zhuang^1,2†^, Chengcheng Li^2^, Huan Zhang^4^, Yuanyuan Liu^5^, Yu Chen^1,2^, Wu Yang^1,2^, Longxi Wu^1,2^, Yawei Du^1^*, Wenguo Cui^1^*, and Hongbo Zhang^1,2,6^*

Affiliations

^1^ Department of Orthopaedics, Shanghai Key Laboratory for Prevention and Treatment of Bone and Joint Diseases, Shanghai Institute of Traumatology and Orthopaedics, Ruijin Hospital, Shanghai Jiao Tong University School of Medicine, 197 Ruijin 2nd Road, Shanghai, 200025, PR China.

^2^ Pharmaceutical Sciences Laboratory, Faculty of Science and Engineering, Åbo Akademi University, Turku, 20520, Finland.

^3^ Departmentof Orthopaedics, The First Affiliated Hospital of Soochow University, Soochow University, Suzhou 215000, China.

^4^ State Key Laboratory of Water Environment Simulation, School of Environment, Beijing Normal University, Beijing 100875, China.

^5^ Institute of Biomedicine, University of Turku, Turku，20014, Finland.

^6^ Turku Bioscience Centre, University of Turku and Åbo Akademi University, Turku, 20520, Finland.

^†^ These authors contributed equally to this work

^*^Address correspondence to: Yawei Du, yaweidu@sjtu.edu.cn; Wenguo Cui, [wgcui80@hotmail.com](mailto:wgcui80@hotmail.com) and Hongbo Zhang, [hongbo.zhang@abo.fi](mailto:hongbo.zhang@abo.fi)

**Supplementary Fig. 1.** Zeta potential of the corresponding nanoparticles at 1, 3, 5, and 7 days.

**Supplementary Fig. 2:** Cumulative release concentration of Zn^2+^ percentage of Mito@ZIF@RGD nanoparticle in buffer solutions at pH 6.5 and 7.4.

**Supplementary Fig. 3.** Time-dependent expression of integrin αVβ3 in RAW264.7 cells during RANKL-induced osteoclast differentiation under E0771 cell treatment. (A) Representative Western blot images showing the expression of integrin αV, integrin β3, and β-actin in RAW264.7 cells at days 1, 2, 3, and 5. (B, C) Quantitative analysis of Integrin αV and Integrin β3 protein levels normalized to β-actin. (D, E) Relative mRNA expression levels of ITGAV and ITGB3 in RAW264.7 cells at days 1, 2, 3, and 5 measured by quantitative real-time PCR (RT-qPCR). Gene expression was normalized to GAPDH and calculated using the 2^^-ΔΔCt^ method. Data are presented as fold change relative to the PBS group. *n* = 3. **p* < 0.05, ***p* < 0.01, ****p* < 0.001, *****p* < 0.0001.

**Supplementary Fig. 4.** RGD modification enhances the phagocytic uptake of differentiating RAW264.7 cells. (A) Differentiating RAW264.7 cells pretreated with free RGD peptide followed by administration of Mito@ZIF@RGD nanoparticles. (B) Differentiating RAW264.7 cells treated with RGE-modified non-targeted nanoparticles (Mito@ZIF@RGE). (C) Differentiating RAW264.7 cells pretreated with an integrin αvβ3 inhibitor prior to Mito@ZIF@RGD administration. (D) Differentiating RAW264.7 cells treated with Mito@ZIF@RGD nanoparticles. (E) Primary BMSCs treated with Mito@ZIF@RGD nanoparticles. Scale bars: 10 and 50 μm.

**Supplementary Fig. 5.** Lysosomal escape of Mito@ZIF@RGD. Colocalization analysis of mitochondria and lysosomes at 0.5 h (upper panel) and 4 h (lower panel), MitoTracker™ Green labels mitochondria, LysoTracker Red labels lysosomes, and Hoechst labels nuclei. Scale bar: 20 μm.

**Supplementary Fig. 6.** JC-1 staining showing mitochondrial membrane potential changes in osteoclast precursor cells under different treatments (green: JC-1 monomer; red: JC-1 aggregate). Scale bar: 50 μm.

**Supplementary Fig. 7.** ROS detection of cancer-associated osteoclast precursor cells following each group treatment. Scale bar : 50 μm.

**Supplementary Fig. 8.** Osteoclast differentiation of cancer- associated osteoclast precursor cells following each group treatment. (A) TRAP staining for osteoclast differentiation. (B) Percentage of TRAP-positive area induced during osteoclast differentiation. (C-E) The mRNA expression levels of osteoclast differentiation-related genes Acp5, Ctsk, and Nfatc1. Scale bar : 50 μm, *n* = 3, *****p* < 0.0001.

**Supplementary Fig. 9.** Bone Resorption pit formation assay of cancer-associated osteoclast precursor cells following each group treatment. (A) The representative images of bone resorption pit formation. The regions outlined by red dashed lines represent the resorption areas. (B) Quantification of resorption pits. Scale bar : 50 μm, *n* = 3. **p < 0.01, ****p < 0.0001.

**Supplementary Fig. 10.** Measurement of ATP levels in cancer- associated osteoclast precursor cells after different treatments. *n* = 3. *p < 0.05, **p < 0.01, ***p < 0.001, ****p < 0.0001.

**Supplementary Fig. 11.** H&E staining of the heart, liver, spleen, lungs, and kidneys in healthy mice after different treatments, scale bar: 100 μm.

**Supplementary Fig. 12.** Serum biochemical and inflammatory cytokine analysis in healthy mice after different treatments. (A-C) Serum levels of ALT, AST, and BUN reflecting liver and kidney function. (D-F) Serum levels of IFN-γ, TNF-α, and IL-6 indicating systemic inflammatory response. *n* = 3.

**Supplementary Fig. 13.** Evaluation of therapeutic outcomes in tumor-bearing mice under different treatment conditions. (A) Tumor growth curves over the treatment period. (B) Serum CTX levels reflecting bone resorption. (C) Serum PINP levels reflecting bone formation. (D) Zinc ion concentrations in joint homogenates. *n* = 5. ***p* < 0.01, ****p* < 0.001, *****p* < 0.0001.

**Supplementary Table 1.** Primer sequences of the genes. (from Sangon Biotech, CN)

| **Gene** |  | **Primer（5’-3’）** |
| --- | --- | --- |
| *Itgav* | Forward | GGTGTGGATCGAGCTGTCTT |
|  | Reverse | CAAGGCCAGCATTTACAGTG |
| *Itgb3* | Forward | CGGGCTAACCGACCAGGTGTCCCG |
|  | Reverse | CCTGCATGATGGCGTCAAAG |
| *Acp5* | Forward | AGACCCAATGCCACCC |
|  | Reverse | GGACCTCCAAGTTCTTATC |
| *Ctsk* | Forward | ATGTGGGTGTTCAAGTTTC |
|  | Reverse | TCAATGCCTCCGTTCT |
| *Nfact1* | Forward | TGGGAGATGGAAGCAAAGAC |
|  | Reverse | ATAGAAACTGACTTGGACGGG |
| *Gapdh* | Forward | TGCCCCCATGTTTGTGATG |
|  | Reverse | TGTGGTCATGAGCCCTTCC |
